# Peripheral blood to next-generation sequencing ready DNA library: a novel engineering design for automation

**DOI:** 10.1186/s12864-024-10892-0

**Published:** 2024-10-22

**Authors:** Dulguunnaran Naranbat, Lothar à Brassard, Nabil Lawandy, Anubhav Tripathi

**Affiliations:** 1 Center for Biomedical Engineering, School of Engineering, Brown University, Providence, RI 02912 USA; 2 Revvity Chemagen Technologie GmbH, Arnold-Sommerfeld-Ring 2, 52499 Baesweiler, Germany

**Keywords:** NGS, Library preparation, Nucleic acid extraction, Peripheral blood, Automation

## Abstract

Whole genome sequencing (WGS) has become a gold standard for diagnosing genomic variation. Peripheral blood is a common sample source for the extraction of nucleic acids for Next-Generation Sequencing (NGS) applications. Here, we present an integrated and fully automated device design that uses new concepts of fluid mechanics, heat-mass transfer, and thermodynamics of enzymatic reactions to extract nucleic acids from the blood and perform DNA library preparation from a pre-filled plate. We demonstrate that the presented device effectively extracts dsDNA with an average of 25.03 µg/mL and 25.91 µg/mL yield from citrate-stabilized human peripheral blood stored in *Fresh* (4 °C) and *Frozen* (-20 °C) conditions, respectively. Furthermore, our method automatically extracts nucleic acids and creates a high-quality sequence-ready DNA library from blood stabilized with citrate and EDTA for 8 samples simultaneously in a single run with a total operation time of ~ 7 h. Our results show the required coverage and depth of the genome, highlighting an essential application of this device in processing blood samples for genome sequencing.

**Graphical Abstract:**

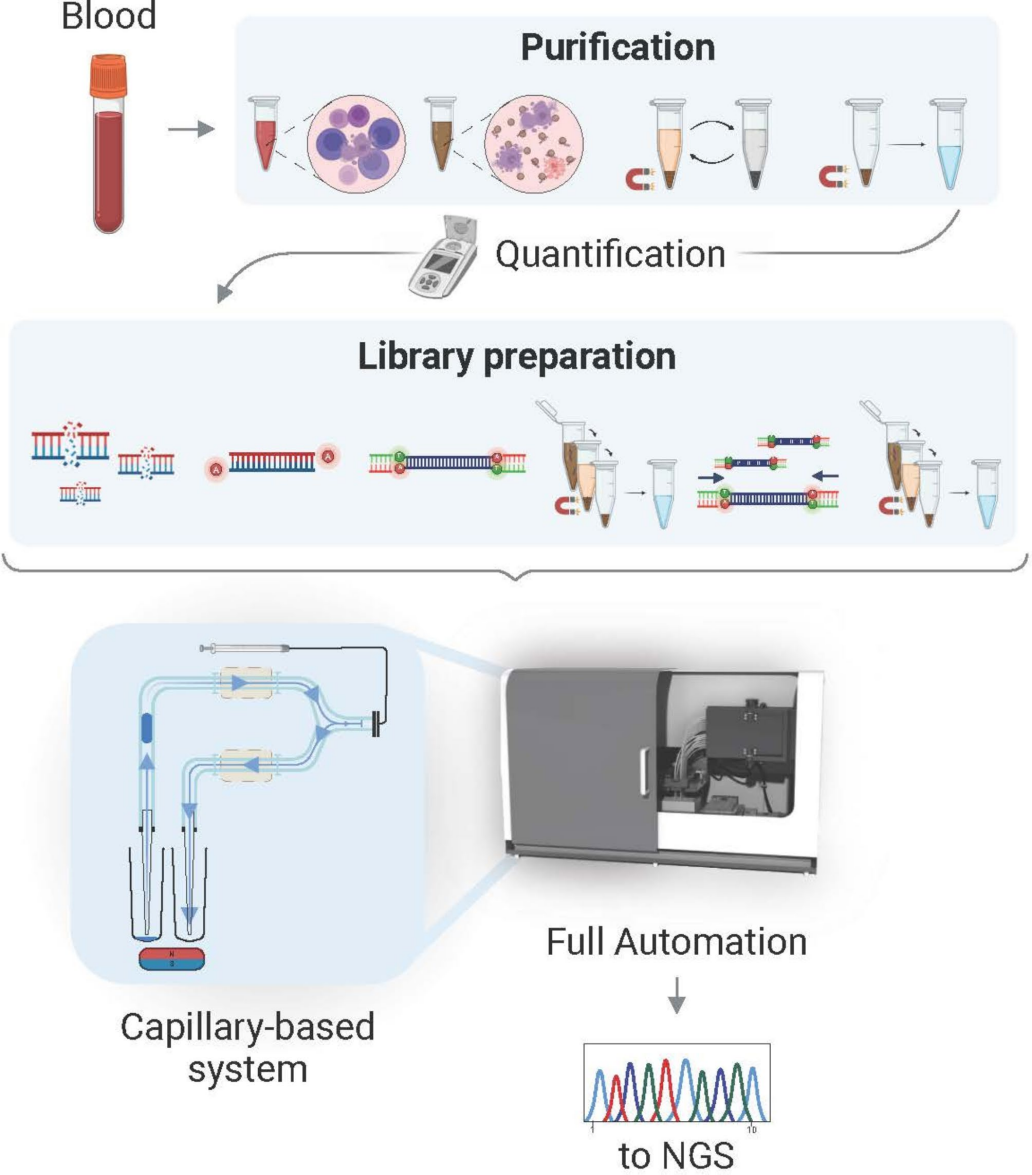

**Supplementary Information:**

The online version contains supplementary material available at 10.1186/s12864-024-10892-0.

## Introduction

Access to sequence-based diagnostics in clinics and research laboratories is essential to advancing the field of genomic studies. Sequencing technology such as Next-generation sequencing (NGS), specifically sequencing by synthesis (SBS), is the leading ‘gold standard’ for whole genome sequencing (WGS) analysis [1]. NGS workflows for biospecimens, such as peripheral blood, require purifying nucleic acid (NA) to prepare pure sequencing libraries, a crucial upstream step before sequencing [2–4]. Conventional manual methods to combine peripheral blood NA purification (NAP) and NGS workflows require multiple steps of pipetting volumes, heating, cooling, vortexing, centrifugation, mixing, and magnetizing [5]. As a result, the entire workflow is time-consuming, labor-intensive, costly, and prone to human errors [6]. Therefore, there is a growing need to automate the preparation of reliable and high-quality peripheral blood sequencing libraries, a desirable workflow for small clinics and research laboratories.

**Fig. 1:**
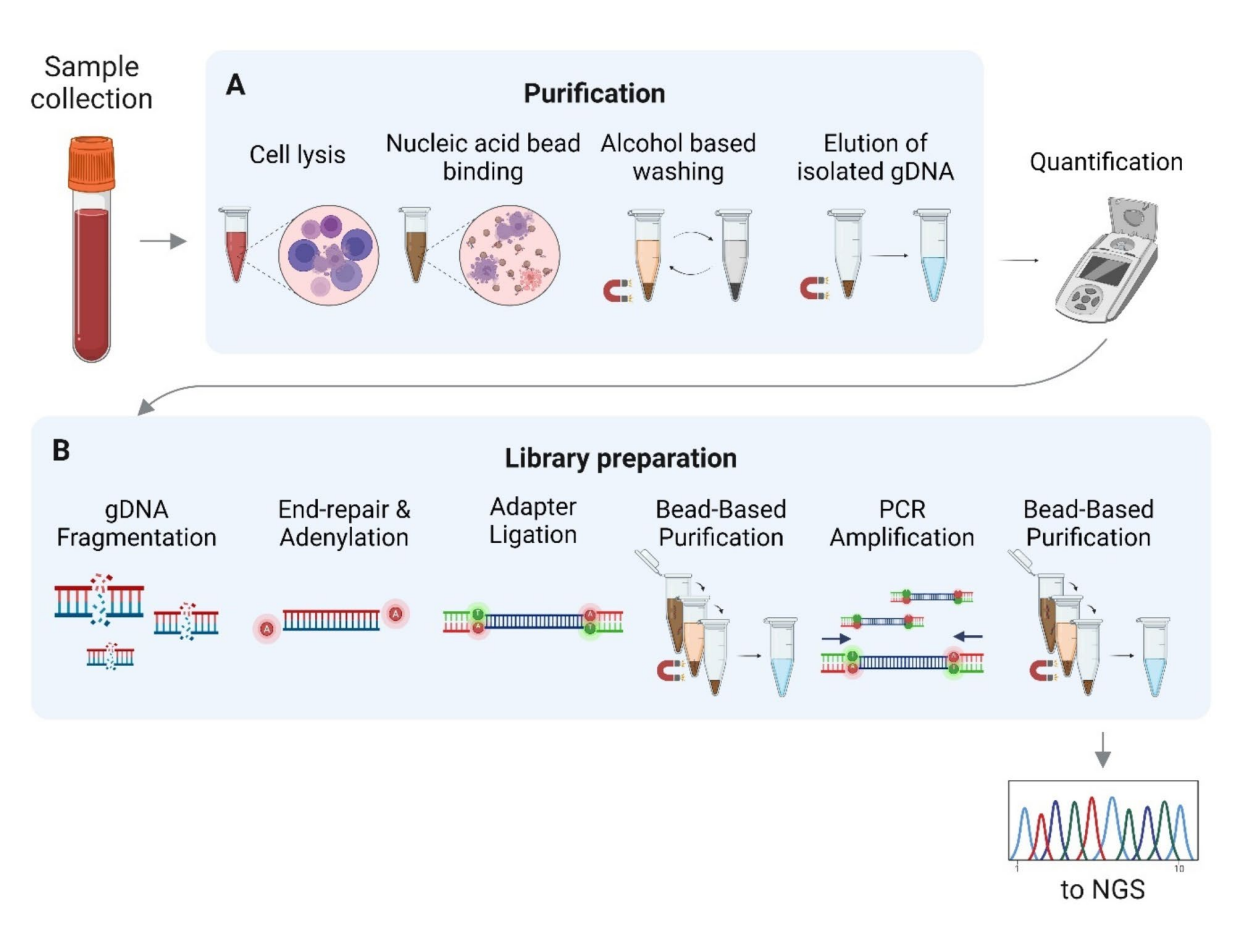
Schematic illustration of the complete sample collection to the NGS workflow. (**A**) Manual extraction of genomic DNA (gDNA) from blood. NA is purified from human peripheral blood by performing multiple mixing and magnetic bead-based purification steps. Optionally, isolated samples are quantified to normalize the output. (**B**) Purified genomic DNA undergoes fragmentation, end modification (repair and adenylation), adapter ligation, post-ligation purification steps, amplification via PCR, post-amplification purification, and library normalization to prepare it for optimal NGS analysis

The two-part conventional protocol, as shown in Fig. 1, requires (1) NAP, which includes lysis, magnetic bead binding, multiple washing, NA elution, and optional QC control to determine extraction yield, and then (2) library preparation, which incorporates genome fragmentation, adapter ligation, post-ligation purification, amplification with polymerase chain reaction (PCR) and post-PCR purification before samples are sequenced. Automating this protocol requires integrating thermal cycling units with robotic arms to move plates, a pump mechanism for volume displacement, motion sensors, a drop tip mechanism, and magnetic zones [7]. Such automation capacities are often only accessible to high-throughput labs, where a large footprint, technical expertise, and a sizable installation and maintenance cost can be sustained. On the contrary, small-throughput local labs are required to work with labor-intensive manual protocols that are more susceptible to variation and errors [8].

The presented automation system is leveraged by integrating fluid mechanics and heat transfer steps to drive the efficiency of biochemical reaction kinetics and molecular thermodynamics while preserving the sample’s integrity with minimum volume loss. Our method relies on (1) capillary-based heating and thermal cycling of sample slugs, (2) a dual fluidic path for reaction plugs to reduce carry-over contamination of enzymes, and (3) a reagent plate design with optimal volumes for reaction mixing. The latter feature also efficiently mixes magnetic beads and DNA purification procedures for the NGS library prep workflow.

Point-of-care (PoC) progress in NAP is mainly driven by efforts to increase the quantity of extracted NAs, reduce the presence of contaminants co-extracted with the NAs, such as RNA, and shorten the steps and duration of sample preparation with higher extraction efficiency. The NGS workflow consists of two main parts: (1) cell lysis and (2) NA isolation and purification for library preparation, where NA isolation can be done using automation-friendly methods such as carboxyl-coated magnetic beads. Once lysed, gDNA can be isolated using a spin column or centrifugal technique; however, purification by carboxyl-coated magnetic beads is more automation-friendly without requiring centrifugal or vacuum force [9–12]. This is due to the ability of the magnetic bead to bind to NA reversibly in a magnetic field instead of requiring centrifugal or vacuum force [13]. Despite the attractive attributes of magnetic beads in automation, much of the success of NAP is limited to the liquid handler’s ability to handle specific volumes with a range of viscosity accurately and precisely, as well as to avoid cross-contamination, reduce bubble formation, and completely resuspend beads.

The purified NAP gDNA will undergo fragmentation before barcoded adapters are added to the ends of the unpolished fragments. Compared to some gDNA fragmentation methods, such as mechanical shear fragmentation, the enzymatic fragmentation method is more automation-friendly since only volume displacement and heating are required for transposases, restriction endonucleases, and nicking enzymes to fragment the gDNA [14]. Unpolished fragments have damaged ends and contain overhangs; therefore, T4 DNA polymerase and Klenow fragments are used to end-repair by blunting fragmented DNA. The T4 polynucleotide kinase (PNK) is used to phosphorylate the 5’ end of the DNA fragment with an overhang of adenine (A), a procedure known as A-tailing [15]. The polished fragment’s A-overhang is ligated complementarily to the Thymine (T) overhang of barcoded indexed adapters to identify specific sample sequences during multiplexing and bind the fragments to a sequencing flow cell [16]. The ligated products are then size-selected to remove any undesirable 120–170 bp-sized adapter dimers. This by-product forms during the ligation step, when two dimers attach to one another and compete for binding sites on the flow cell, eventually reducing the efficiency and quality of the sequencing [16]. Size selection can be made using solid phase reversible immobilization (SPRI) magnetic beads-based isolation, where the ratio of polyethylene glycol (PEG), salts, and magnetic particles are balanced to select > 200 bp fragments from the input material [17]. The purified polished fragments are amplified using a standard PCR procedure targeting sequences in the adapters and then purified to be analyzed for sequencing.

**Fig. 2:**
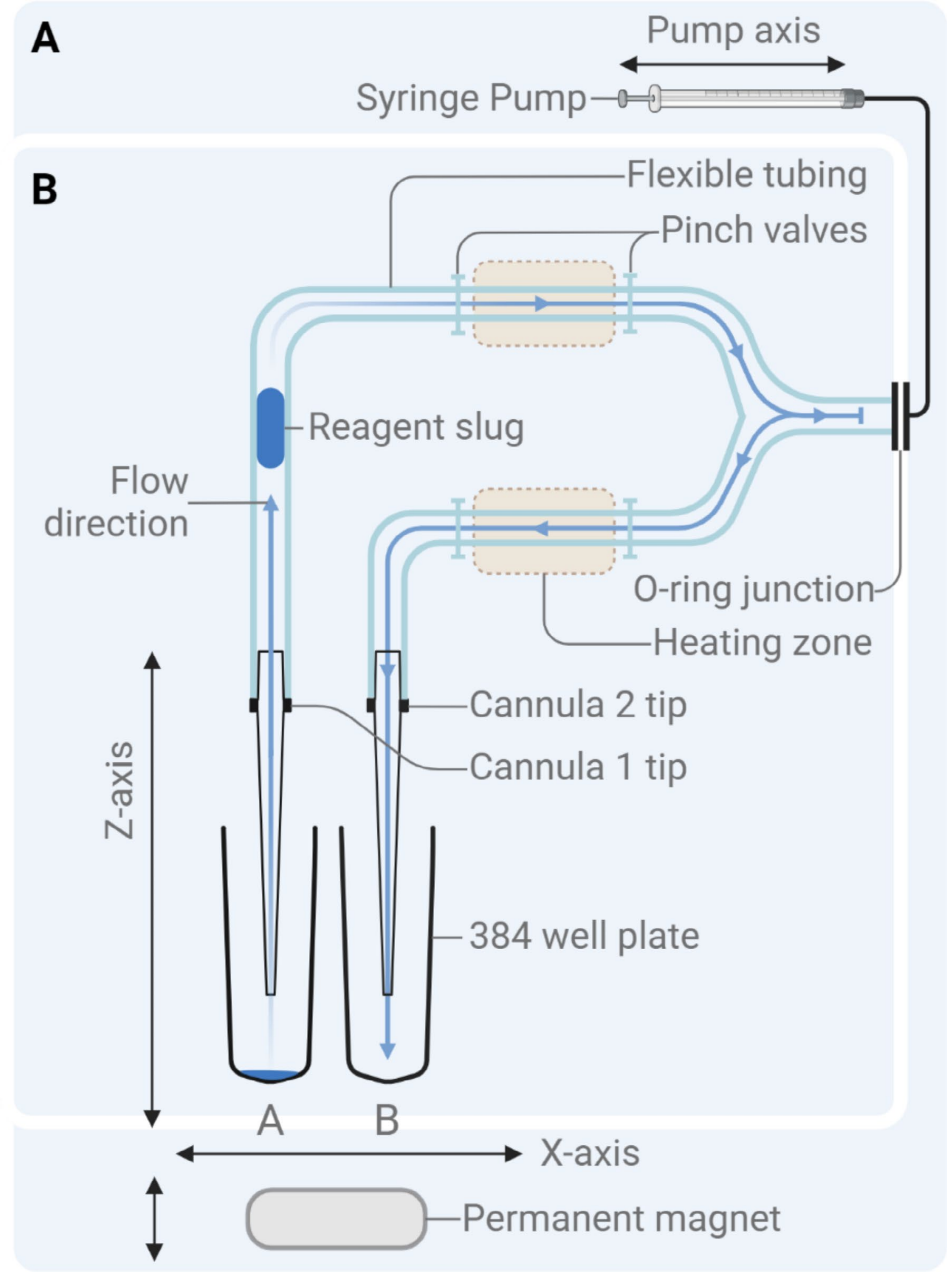
Schematic view of the principles of liquid handling of the automation system. (**A**) The automated system with a permanent magnet, syringe, and tubing to manipulate liquid reagents and magnetic particles. (**B**) Single-use custom-built cartridges and 384-well plate consumables. The cartridge cannula tips are moved along the Z axis and the X axis to handle liquid, while a permanent magnet is placed under the 384 plate wells, also moved along the Z axis. The reagents move unidirectionally from one cannula to another in the Y-junction to avoid contaminated areas. The slugs are thermally treated in the heating zone with valves to contain and pressurize reagents during thermal incubation

The presented automation system utilizes flexible tubing to handle liquid during the nucleic acid extraction and library preparation steps. The innovative design of the flexible tubing allows capillary-based thermocycling while simultaneously accessing eight runs in two separate rows of wells (Fig. 2). The flexible tubing is pinchable, allowing the isolation of reagent slugs at a consistent temperature and pressure. The custom design of the two-cannula system provides reagents to move in a unidirectional motion without revisiting any contaminated areas. The cannulas are controlled along the Z-axis to move the tip to specific volume heights accurately.

**Fig. 3:**
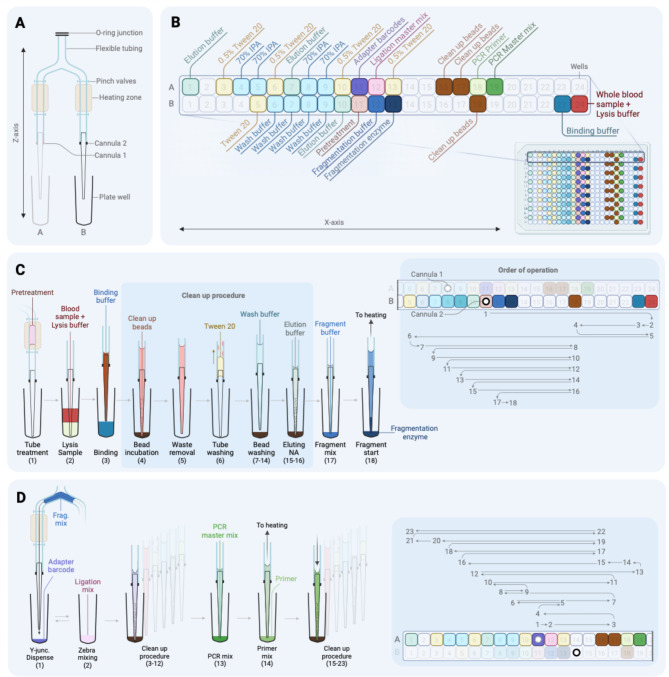
Schematics of the consumables and the plate layout. (**A**) Overview of single-use consumable flexible tubing with a Y-junction connecting two cannulas. (**B**) Plate layout of the reagents on a consumable 384-well plate. (**C**) NAP process with fragmentation, including the cleanup procedure with specialized order of operations in Lane B/Cannula 2 (right blue box). (**D**) Library preparation process after the fragmentation of extracted gDNA with a separate order of operations in Lane A/Cannula 1 (right blue box)

All reagents are positioned to restrict any carryover or cross-contamination between samples, as seen on the plate layout, and are accessible by the cartridge from a single plate during the entire run (Fig. 3A). The alternating rows of 384-well store all necessary reagents, Lane B for NAP and Lane A for library preparation (Fig. 3B). The order of operations for optimal fluid transport is significant for the cascade of reactions during both procedures; hence, the order of operation for NAP and library preparation is shown in Fig. 3C and D. The initial user interaction for setup is ~ 15–20 min for plate loading, and the whole purification and library preparation process is automated in a run time of 7 h. Methods for automating and integrating complete preparation of the NGS library from human peripheral blood samples are discussed further in this study.

## Materials and methods

### Mechanical design for an automation system

The automation device can handle liquids, magnetic bead isolation, and thermocycling to run a complete sample to sequencing-ready products for NGS. The instrument comprises eight individual syringe pumps (Hamilton, Reno, NV, USA) for each sample lane, an X-stage 384 plate holder, a magnetic bar (K&J Magnetics, Pipersville, PA, USA) under the X-stage, a custom-made cannula boat holder, and four separate pinching valves – all with individual stepper motors (Lin Engineering, Morgan Hill, CA, USA; Figure S1). Motors are dictated by a custom-built local controller board and custom library instrument control software. In addition to the device, two consumables are used in every experimental run – a custom-built cartridge for liquid handling and a deep well 384-well plate to store the reagents. Individual syringe pumps drive the displacement of the liquid in the cartridge.

A custom heating element and pressurization mechanism design control temperature and provide efficient thermocycling in the library preparation procedure. The heating element is composed of three components: (1) a copper plate grooved to match the contour of the tube with direct thermal contact, (2) two Peltier modules (Digikey, Thief River Falls, MN, USA) at 40 × 40 mm size to drive heating, and (3) a heat sink attached on the opposite side with a fan (Digikey, Thief River Falls, MN, USA) to dissipate excess heat and maintain constant desired temperature (Figure S1). A detachable door with insulation padding (Digikey, Thief River Falls, MN, USA) ensured that the tubing had complete surface contact with the heating element. A custom-designed printed circuit board (PCB) controlled the motors, the Peltier, and the fan. The temperature control was regulated by a thermocouple inside a tubing that was permanently placed on the heating plate. Heat driving is achieved through a custom hybrid PID, where the Astrom-Hagglund method implements the most optimum settings.

The complete occlusion of the liquid in the heating area of the cannula is essential during heating to control the evaporation rate. There are a total of 4 custom triangular pinching valves that are positioned outside each capillary tube column (2 valves in each column). Each valving is controlled by a stepper motor that extends out to pinch the flexible tubing against the removable door during heating and pressurization; each valving is responsible for pinching eight cannula tubes at a time. The physical specification of the device measures 28.58 cm x 41.91 cm x 66.04 cm.

### Design innovation for the consumables

The consumable cartridge for liquid handling is composed of four components (Figure S2) – (1) a total of 16 RoboRack P30 MDT sterile pipette tips (Revvity, Waltham, MA, USA) where the liquid is in contact, (2) a custom-designed plastic boat holder to stabilize the 16 cannula tips in two parallel alignments, (3) flexible capillary thermoplastic Flexelene tubing without PVC or DEHP for liquid displacement (Eldon James, Fort Collins, CO, USA) and (4) custom-built plastic casing to immobilizing the plastic tubing in place during heating and creating airtight seal junction to the airway connected to the syringe pump (O-ring junction). Custom-carved copper plates are placed behind the tubing to provide the most surface area for maximum heat transfer. The junction between the lines and the cartridge is sealed using an elastic O-ring, allowing each consumable to be easily interchanged per run without changing the airlines or the syringe. The capillary tubing is malleable and flexible and prone to complete pinching; this is important during pressurization and heating. Each capillary tubing is airtight sealed to a corresponding 16 lanes of pipette tip, arranged in two separate columns. The spacing between columns is 4 wells (384-well plate unit). Each cannula column is attached in a Y-junction, allowing the liquid to pass from one tip to another unidirectionally. The end of the Y-junction is where the consumable and the O-ring meet from the instrument. The cannula tips with the tubing are fixed perpendicular to the Z axis and can only move vertically in an up-and-down motion. The fixation of the cartridge along the Z axis is supported by the cannula boat holder, where each cannula column is positioned in an alternating zigzag fashion (Figure S3). Each column of the cannula has 8 individual plastic cannula tips, where the cannula tips can access the wells A, C, E, G, I, K, M, and O at column 1 and the wells B, D, F, H, J, L, N, and P at column 4 of a 384-well plate – this is to allow each cannula tip to enter uniquely new well without contaminating the pipette tips within a run. The entire cartridge assembly is a replacement for the conventional drop-tip pipetting mechanism.

The other consumable is a sterile Thermo Scientific Nunc DeepWell 384-well plate (Thermo Fisher Scientific, Waltham, MA, USA). Two rows of wells correspond to a single sample run, and eight samples can be run simultaneously in each run. The consumable 384-well plate is placed on the X-stage of the instrument and moves along the X-axis horizontally. The magnet directly under the X stage is used to pellet MNPs at the bottom of the 384-well plate during the washing steps of NA purification. Select sequences of motor motions in the stated axis are scripted in different algorithms to handle the liquid best and isolate the beads during washing and NA purification procedures. These algorithms are revised, structured, and communicated to the PCB using Python programming codes to control the respective motors. The percentage of volume loss experiment during volume displacement (200 mm) in the tubing was measured by gravimetric LB-200-124e analytical balance (Cole-Palmer, Vernon Hills, IL, USA). Prevention of splash carryover from lane-to-lane or extended evaporation is possible by adhering a Pre-slit Adhesive Sealing Film (Analytical-sales and services, Flanders, NJ, USA) to the 384-well plate.

### Sample collection and ethical consideration

Citrate and EDTA stabilized human peripheral blood samples were collected and purchased from UKA Blutspendedienst, University Hospital RWTH Aachen (Uniklinik RWTH, Aachen, Germany). Sample collection followed standard blood donation protocol according to the ethical standards of the Declaration of Helsinki (approved by the ethics committee of the University Hospital Aachen, RWTH University, Aachen, Germany) [18]. Standard informed consent forms were collected from patients, samples were anonymized, and no personal information was provided about donors during the study [19].

### Sample storage and processing – NAP, qPCR & NGS library preparation

The automation system used the chemagic Body Fluid 1k Kit (Revvity chemagen Technologie GmbH, Baesweiler, Germany) to purify NA. The study evaluated two distinct sample preservation methods before analysis: *Fresh* samples in which peripheral blood was maintained under refrigerated conditions of 4 ° C and processed within the time frame of 1 to 7 days post-collection. *Frozen* samples where peripheral blood was frozen at -20 °C for a minimum duration of 48 h after the initial collection. Repeated freeze-thaw conditions were completely avoided. The manufacturer’s user guide was used to perform the manual extraction process. The automation system used Lysis buffer at 40 µL, Binding buffer at 120 µL, Wash buffers 2–6 at 200 µL, and elution buffer at 30 µL. A safe volume of 10% increase was incorporated. Carboxyl functionalized magnetic beads ($\:140\frac{mg}{mL}$) at varying volumes were used to determine the best binding capacity. Additionally, 200 µL of 0.5% Tween-20 wash solution (Bio-Rad Laboratories, Hercules, CA, USA) was used to clean the flexible tubing between the Lysis, Binding, and Wash steps. The NAP workflow followed the procedure illustrated in Fig. 3C. For the other automation systems – Prepito and chemagic 360 (Revvity chemagen Technologie GmbH, Baesweiler, Germany), a Body Fluid 1k Kit designated for each of the instruments with 400 µL input human blood sample and 200 µL extraction volume was used for the extraction method using the manufacturer’s corresponding script protocols.

The purified NA in elution buffer was then processed to test qPCR quality targeting the housekeeping reference ALB gene [20]. 5 µL of purified template DNA was mixed with the final concentration of 1X SensiFAST Probe Lo-ROX Kit Mastermix (Bioline, London, UK), 0.4 µM forward ALB primer, 0.4 µM reverse ALB primer, and 0.4 ALB probe with FAM tag (Biomers, Ulm, Germany) for a total of 20 µL reaction. Thermocycling and detection were completed with the QuantStudio 5 real-time PCR system (ThermoFisher Scientific, Waltham, MA, USA).

30 µL of eluted gDNA was used as the template for the library preparation using the Watchmaker DNA Library Prep Kits with Fragmentation (Watchmaker Genomics, Boulder, CO, USA) with NEXTFLEX Unique Dual Index Barcodes (Revvity, Waltham, MA, USA) at 4 PCR cycles. In addition to the manufacturer’s user guide for library preparation, a custom-designed pretreatment solution is used to decrease the adsorption capacity of the flexible tubing at the beginning of the procedure to prevent any independent adsorption of NA of interest. The coating of the pretreatment solution is composed of Tris buffer conditioner pH 7.5 (ThermoFisher Scientific, Waltham, MA, USA), MgCl_2_ (Sigma-Aldrich, St. Louis, MO, USA), Dithiothreitol (ThermoFisher Scientific, Waltham, MA, USA), 10% Tween-20 (Bio-Rad Laboratories, Hercules, CA, USA), Bovine serum albumin (Sigma-Aldrich, St. Louis, MO, USA), and custom dideoxynucleotides at ~ 20bps (Integrated DNA Technologies, Coralville, OH, USA). To determine the capacity of the pretreatment solution, 40 ng of male human genomic DNA input (Promega, Madison, WI, USA) was used. To prevent extended evaporation, ultrapure nuclease-free water (Integrated DNA Technologies, Coralville, OH) was added to low-volume reagents such as fragmentation buffer, fragmentation enzyme, barcode adapters, and primers for PCR on the 384-well plate at respective volumes of 3 µL, 3 µL, 5 µL, and 10 µL. Volumes were chosen according to the total volume handled during heating and the time elapsed since the start of the protocol to compensate for the estimated evaporation rate. Similarly, to compensate for additional evaporation, 70% isopropyl alcohol (IPA) was used for the washing step. The NGS library preparation workflow followed the procedure illustrated in Fig. 3D. The complete sequence of logic for the liquid displacement of all reagents can be observed using the order of operation’s chronological order (1–18 for NAP and 1–23 for library preparation) as shown in the right blue box of Fig. 3C and D.

### Analysis and quality control

All purified NAs were analyzed using the Agilent BioTek Gen5 microplate imager (Agilent, Santa Clara, CA, USA) to assess the quality of the NA (260 nm / 280 nm). In contrast, the Fisher Scientific Qubit Flex Fluorometer with the High Sensitivity DNA assay (Invitrogen, Waltham, MA, USA) was used to assess the quantity of the dsDNA. Comparison of NA purification workflows was analyzed using a two-way ANOVA test with the Turkey test as a multiple comparison analysis with R scripts (R Foundation for Statistical Computing, Vienna, Austria). Illustrations and comparison graphs were created with BioRender.com (BioRender, Toronto, Ontario, Canada), Adobe Illustrator (Adobe, San Jose, CA, USA), and Microsoft Excel (Microsoft, Seattle, WA, USA).

The prepared libraries were accessed on the LabChip GX Touch (Revvity, Waltham, MA, USA) nucleic acid analyzer and the 2100 Bioanalyzer Instrument (Agilent, Santa Clara, CA, USA) for size range and output yield before running a complete sequencing assay. Data generated on the 2100 bioanalyzer instrument was further assessed using the bioanalyzeR package [21]. All samples were sequenced using the MiniSeq platform at a 2 × 150 bp sequencing rate (Illumina, San Diego, CA, USA). The generated data were further processed and visualized using custom R scripts (R Foundation for Statistical Computing, Vienna, Austria): Bowtie2, Trimmomatic, Picard, RColorBrewer, gridExtra, Samtools, tidyverse, ggplot2, scales, and reshape2. All the analyses of the automated workflow and manual workflow were compared here.

## Results

### Preconditioning of flexible tubing (pre-NAP)

The overall liquid handling capacity of the automation system is based on the capabilities of the flexible tubing, such as its ability to accurately and precisely displace reagents and withstand high-temperature cycling. While the cannula tubing provides a unique method of handling liquid, the inner surface of the tubing adsorbs active biomolecules such as NA and active enzymes due to its active thermoplastic elastomer (TPE) composition, making it non-ideal for downstream applications [22, 23]. Hence, tube preconditioning must be performed before the NAP steps (Fig. 3C step 1) for the subsequent library preparation process. A preconditioning solution was developed to occupy the adsorption sites on the surface of the tube and make it less porous through the coating.

**Fig. 4:**
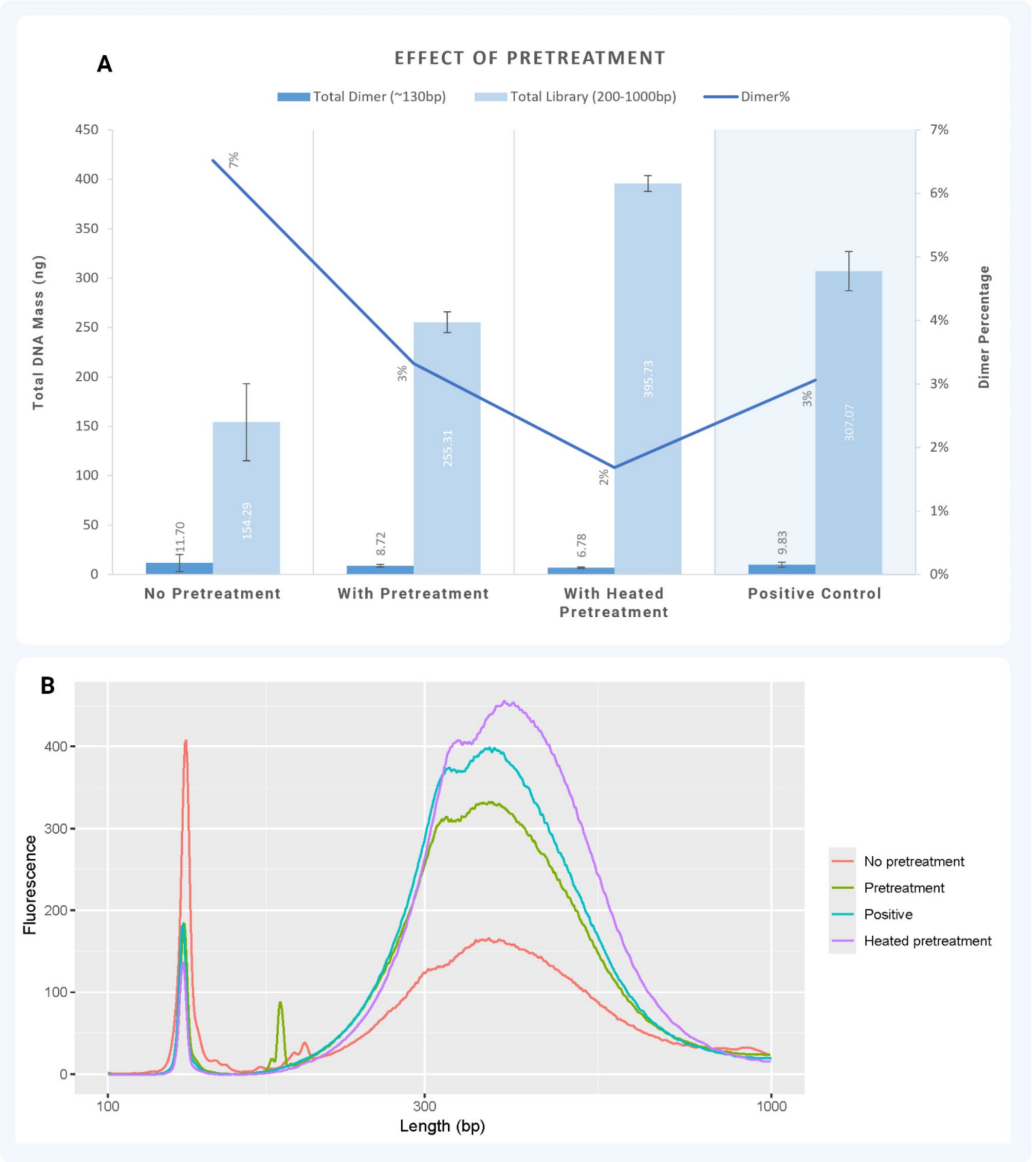
Analysis of capillary tubing preconditioning. (**A**) Effect of pretreatment on the yield of total library mass, adapter dimers, and dimer percentage compared with all treatment conditions against the control (manual library preparation). (**B**) Overlaid visualization of the library size distribution electropherogram and the adapter dimer’s peak intensity

**Fig. 5:**
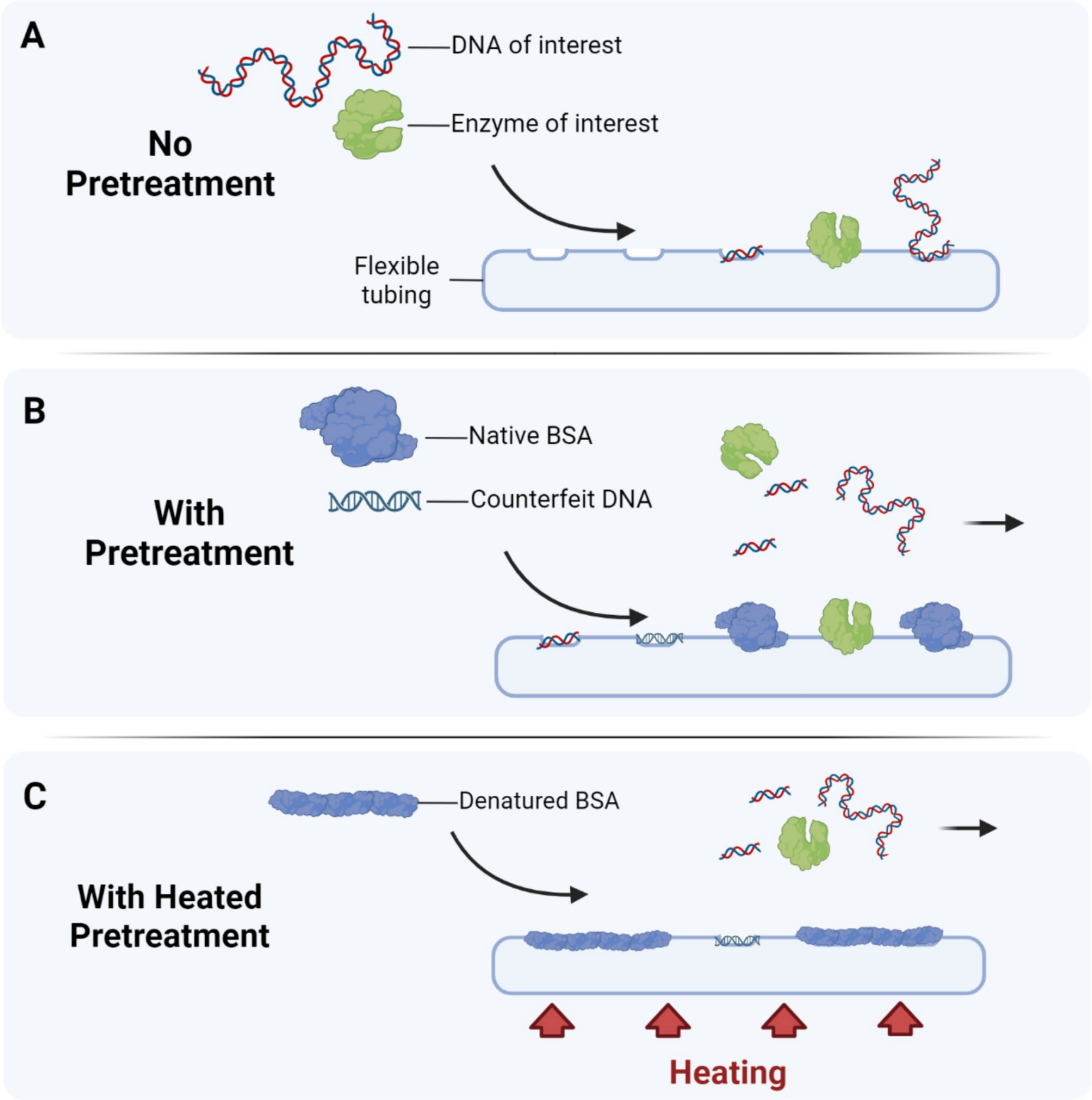
Schematic illustration of pretreatment adsorption with flexible tubing. (**A**) No pretreatment conditions where DNA of interest (gDNA/fragmented DNA/adapters) and enzyme of interest (Fragmentase/end-repair and adenylation tailing enzyme mix/ligase) are adsorbed to the surface. (**B**) Pretreatment condition where native BSA and short DNA oligos adsorb the inner tubing affinity pores. (**C**) Heated pretreatment conditions where the pretreatment is aspirated into the heating zone to denature the native BSA to increase surface area for more efficient adsorption of the sites

Library preparation experiments were performed using a total of 40 ng of gDNA input to quantify and compare the effect of preconditioning on standard processes. The results in Fig. 4A show that nonspecific adsorption of enzymes and NA led to a ~ 50% reduction in library yield and ~ 20% increase in adapter dimers compared to manual benchtop library preparation (*control*), indicating loss of library prep reagents and input materials. Alternatively, room-temperature pretreatment conditioning the tube surface resulted in a ~ 15% reduction in library yield and a similar percentage of dimers (3%) compared to the control. Finally, the preconditioning with the heated step resulted in a ~ 20% increase in library yield and a 2% total dimer percentage compared to the control with a 3% total dimer. The results in Fig. 4B show the corresponding electropherogram plots for the effect of preconditioning on the quality and size of libraries. Bovine serum albumin (BSA), polysorbate 20 (Tween-20), and inactivated double-stranded DNA (dsDNA) short oligos (Fig. 5) were active components of the pretreatment solution in coating the tubing inside. BSA and Tween-20 molecules reduce surface tension and non-specific binding of molecules to the tubing surface [24]. dsDNA oligos were designed to preadsorb at sites prone to charge-based interactions (Fig. 5B) [25–27]. Heat-induced denaturation of BSA irreversibly unfolded and oligomerized the proteins, resulting in a more adhesive oligomeric form that binds to the elastic tube (Fig. 5C). Similarly, Park et al. (2018) demonstrated that the blocking efficiency of BSA can be increased ten times when treated with heat [28]. In the automation system, the pretreatment solution was heated at 65°C for 5 minutes and then cooled to room temperature before dispensing. Additionally, the inactivated dsDNA short oligos helped to occupy adsorption sites with more affinity, thus minimizing the adsorption of gDNA or fragmented DNA during the library preparation steps. Modified dideoxynucleotides (ddNTPs) were added to the 3’ prime end of the short oligos, preventing any 3’ prime end extension by DNA polymerases and preventing the oligos from interfering with sequencing quality and post-analysis.

The findings of our study revealed that the quality and quantity of libraries prepared using flexible tubing are initially lower than those prepared using a pipette-based workflow (manual library preparation). However, this disparity is significantly reduced when a preconditioning solution is used as a pretreatment for the tubing, especially when heated, providing a solution to enhance library preparation efficiency when using flexible tubing.

#### Fluid slug heating in the flexible tubing

As previously presented, heat transfer to fluid slugs is required in the preconditioning process (Fig. 3C) and the fragmentation and PCR steps (Fig. 1and Fig. 3). The flexible tubing (Fig. 2) was sandwiched between a custom-built grooved copper plate as the heating surface (Figure S1) and a heating pad to effectively conduct heat with an increased contact surface area. The desired temperature of the fluid slugs in the tubing is attained by heat transfer through the copper plate on one side of the tubing. The opposite surface area of the tubing is insulated with a heating pad installed on a detachable door. Once placed on the heating surface, the door presses against the flexible tubing (Figure S2) when closed. Since the heat is only applied from one side, the assumed steady-state one-dimensional conductive heat flow rate through the cylindrical tube wall (*Q*) can be estimated using the following Eq. (1):

1 $$Q = k\pi rL\left( {\frac{{\Delta T}}{{\Delta r}}} \right)$$

where $\:\varDelta\:r=0.8\:mm$ denotes the thickness of the tubing wall, $\:\varDelta\:T=65^\circ\:\text{C}$ denotes the temperature difference between the outer and inner surfaces (the conductive resistance from 25 °C to 90 °C) of the tube in an average radius of $\:r=1.2\:mm$, $\:L\:\cong\:27\:mm$ denotes the length of the slug and $\:k\cong\:0.4\frac{W}{mK}$ denotes the thermal conductivity of the polyethylene tube [29]. Similarly, the rate of conductive heat flow inside the fluid slug itself was estimated using the same Eq. (1), where $\:k\cong\:0.598\frac{W}{mK}$ (thermal conductivity of water-based reagents) [30]. The heat flow rates were approximated to match the rapid steady state between the tube, $\:{Q}_{t}\cong\:3.31\:W$, and fluid slugs, $\:{Q}_{s}\cong\:3.30\:W$ ensuring consistent heat transfer. The cylindrical shape of the fluid slug itself enhanced the heat transfer time across the cross section, and the thermal diffusion time ($\:{t}_{d}$) was estimated using the following Eq. (2):

2 $${t_d} = \frac{{{r^2}}}{{2\alpha }}$$

Here, $\:\alpha\:=0.14558\frac{m{m}^{2}}{s}$ is the thermal diffusivity of the water-based reagents and $\:r=0.8\:mm$ is the radius of the fluid slug [31]. The estimated thermal duration is around 2.20 s, representing the characteristic time it takes for a change in thermal energy to propagate to the center of the fluid slug due to heat flow [32].

Furthermore, the thermal capacitance of the fluid slug is estimated using the following Eq. (3):

3 $$q = \rho \pi {r^2}L{C_s}\Delta T$$

The mass of thermal capacitance is estimated from the mass, $\:m={\uprho\:}{\uppi\:}{\text{r}}^{2}L$ where the density of the plastic is $\:{\uprho\:}=\:0.925\frac{\text{g}}{{\text{c}\text{m}}^{3}}$ [33], radius of the fluid slug is $\:r=0.8\:mm$, the length of the fluid slug is $\:L\:\cong\:27\:mm$, and the specific heat capacity of water-based reagents $\:{C}_{s}=4.2\frac{J}{g^\circ\:C}$. The thermal capacitance of the fluid slug is $\:q\:\cong\:13.7\:J$, where $\:\frac{q}{{Q}_{s}}=4.16\:s$ provides the time constant to heat the entire fluid slug due to its heat storage capacity [34]. Therefore, the time constant of resistive heat transfer (4.16 s) is larger than the thermal diffusion time (2.20 s), which indicates a faster internal heat distribution once the heat is introduced. However, when subjected to a heat source, the fluid takes a longer heating time to reach its final temperature.

To maximize the heat transfer rate during fluid heating, the air surrounding the front and back ends of the slug was compressed by pinching the tubing at the opposite ends of the tubing with the instrument’s pinch valves and before pressurizing with the syringe pump (Fig. 2). The pressurization procedure is illustrated in Figure S4 B-C. This provided consistent heat transfer across the copper-polyethylene tubing and polyethylene tubing-fluid slug interfaces by expanding the flexible tubing and increasing internal pressure (further discussed in *Optimization of Library Preparation Steps*).

A custom proportional, integral, and derivative (PID) equation regulated the copper temperature with a permanent thermocouple placed inside a tubing on the heating zone. The standard error in temperature was measured to be 0.025 °C, 0.319 °C, 0.283 °C, 0.237 °C, 0.155 °C, -0.041 °C, 0.250 °C, and 0.187 °C across the set points of 30 °C, 40 °C, 50 °C, 60 °C, 70 °C, 80 °C, 90 °C, and 100 °C, respectively (as shown in Figure S5 and Figure S6). The ramp rate to heat the inside of the tube was different depending on the desired temperature (as shown in Figure S7). The maximum heating rate was observed between 30 and 40 °C, and as the desired temperature was set higher, the ramp-up rate slowed proportionally. The average ramp rate from 30 °C to 100 °C was $\:0.63\frac{{{}^ \circ C}}{s}$ while the cool-down rate was $\: - 1.02\frac{{{}^ \circ C}}{s}$The measured temperatures guided all the other fluid slugs’ heating steps in library preparation and showed reliable heat transfer.

#### Fluid slug motion

**Fig. 6:**
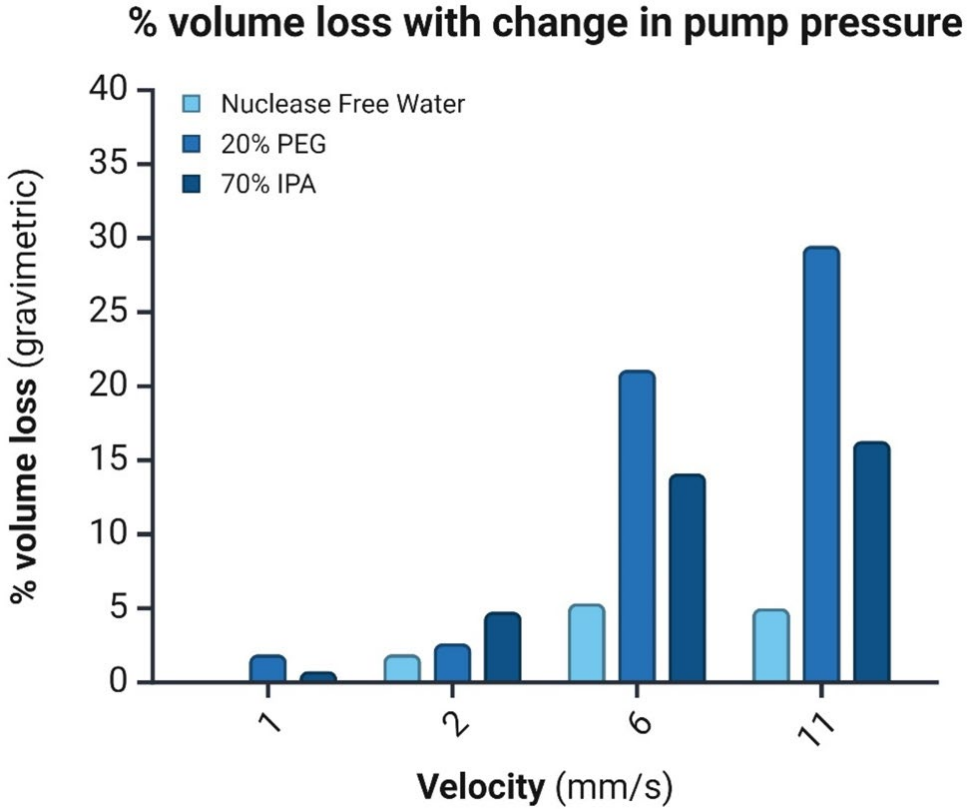
Total percent volume loss of nuclease free water, 70% IPA, and 20% PEG in flexible tubing against the change in pump velocity corresponding to the slug speed inside the tubing. Using a faster speed to move the pump syringe changes the fluid slug morphology, leading to volume loss against the inner surface of the tubing due to droplet formation and film layer formation from the trailing edge of the slugs. In a unidirectional and slower movement (1 $\:\frac{\text{m}\text{m}}{\text{s}}$) the volume was lost minimally (< 2%) (*n*=4)

The integrity of the fluid slug during its motion to and from the heating section was observed at various velocities. A homogeneous and continuous slug motion is vital in library preparation for enzymatic kinetics and liquid transfers. Any loss of liquid volume and breakage or compartmentalization of the slugs resulted in incomplete or inefficient reactions downstream. Figure 6 shows the measured volume loss of fluid slugs (nuclease-free water, 70% IPA, 20% PEG 8000) for various slug speeds, where high- and low-viscosity reagents have greater percentage volume loss at a higher velocity of slug motion. The three different fluids were chosen because they are similar to the reagents used in the NAP and library preparation workflow. The observed volume loss was correlated with the number of capillaries. Here, the capillary number (*Ca*) of the slug motion is estimated using Eq. (4):

4 $$Ca = \frac{{\mu U}}{{{\gamma ^{sl}}}}$$

The estimated capillary number is dependent on fluid viscosity (µ), fluid velocity (U), and surface tension of the solid and liquid boundary ($\:{\gamma\:}^{sl}$). Young’s equation is used to estimate $\:{\gamma\:}^{sl}$ as follows (5):

5 $${\gamma ^{sl}} = {\gamma ^s} - {\gamma ^l}cos\theta$$

Based on the solid surface tension ($\:{\gamma\:}^{s}$) of $\:30\:mN/m$ for Polyethylene [35] and approximated liquid surface tension ($\:{\gamma\:}^{l}$) and the contact angle ($\:\theta\:$), the capillary numbers of each fluid slug tested were estimated accordingly in Table 1.

**Table 1:** Capillary numbers for fluid slug motion in three different fluids used in the automation system

Velocity of fluid slugs (U) [mm/s]	IPA (70%)	Water	PEG 8000 (20%)
1	1.00E-04	2.06E-05	1.14E-04
2	2.01E-04	4.12E-05	2.28E-04
6	6.03E-04	1.24E-04	6.85E-04
11	1.11E-03	2.27E-04	1.26E-03
Viscosity (µ) [mPa*s]	1.72 [36]	0.89 [37]	9.7 [38]
Liquid surface tension (γ^l^) [*mN/m*]	24.47 [39]	72.8 [40]	58 [41]
Contact angle (θ) [°]	45 [42]	96 [43]	104 [44]

At a significantly increased velocity ($\:\text{U}>\:6\frac{\text{m}\text{m}}{\text{s}}$), the fluid slugs disintegrated into multiple different liquid droplets, changing the morphology of the fluid and resulting in a significant volume loss (Fig. 6). At lower velocities ($\:\text{U}<\:6\frac{\text{m}\text{m}}{\text{s}}$), the fluids had 100% slug continuity and integrity of the slug with less than 5% volume loss. The results from Table 1; Fig. 6 show that the approximated capillary numbers have a similar trend to the observed percentage volume loss, where > 10% of the volume is lost when the capillary number surpasses ~ 5.0E-04. The correlation between capillary number and volume loss percentage was linearly correlated at R [2] = 0.879 (Figure S8).

**Fig. 7:**
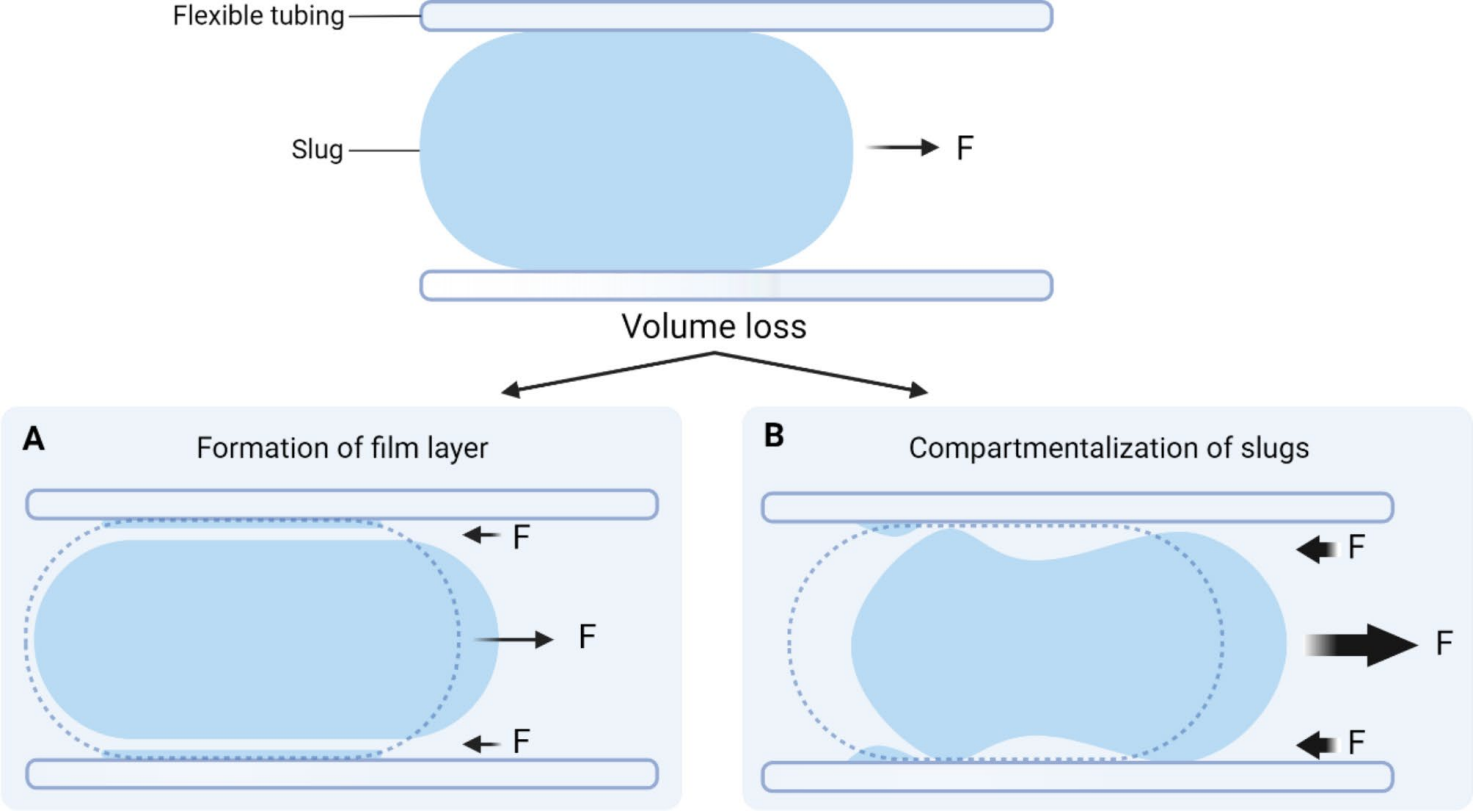
Schematic illustration of volume loss during slug movement due to the change in pump and fluid velocity of the slug. Two mechanisms of volume loss are proposed: (**A**) through the formation of a film layer due to the intermolecular force of the plastic tubing, and (**B**) through compartmentalization of the slugs, where droplets are formed and left from the trailing back edge

There are two proposed mechanisms of volume loss from our observation (Fig. 7): the formation of a film layer against the inner surface of the tube and the compartmentalization of slugs during a large displacement area. When the velocity is $\:\text{U}<\:2\frac{\text{m}\text{m}}{\text{s}}$, the slug’s fluid elements of the slug remain intact, preserving any significant volume loss. However, as more distance of tubing is traveled, the slug inherently creates a surface tension that adheres volume to the inner tubing. The vector force of the slug’s movement direction is opposite to the surface due to tension leaving the volume loss sheet layer (Fig. 7A). When this vector force of slug movement increases, the force of surface tension in the opposite direction proportionally increases, leading to air pockets forming when the slug stream’s morphology changes. This leads to the compartmentalization and breaking of slugs into droplets and bubbles of different sizes, ultimately resulting in volume loss (Fig. 7B). The observed fluid slug motion navigated our script protocols for best-optimized liquid handling processes, where appropriate slow speed was used to minimize volume loss inside the tubing.

### Extraction of genomic DNA from blood

The extraction of genomic DNA from peripheral blood consists of multiple steps (Fig. 1A), including lysis of the blood sample cells, binding of NA to magnetic beads with the aid of binding buffer, purification of NA by washing unwanted substrates, and finally, elution of isolated NA from magnetic beads (Figure S9 and Figure S10).

Detergents in the lysis buffer initiate the breakdown of white blood cells and cell nucleus membranes. Their amphipathic nature allows them to disrupt the lipid bilayers of cell membranes while denaturing the nucleases’ structure [45–47]. Here, the success of cell disintegration is dependent on the ratio of lysis detergent to the input content of peripheral blood and the degree of homogenization during mixing (Fig. 3C **step 2**). Increased homogeneity was achievable with at least one complete cycle of the Depelleting Jet (DJ) mixing technique consisting of 5 iterations of *Fast Mix* (aspirating from the bottom of the plate well then jet dispensing at high speed) for every 1 iteration of *Cyclic Mix* (dispensing specified portion of the aspirated volume at the bottom of the plate well before dispensing the remaining volume above the liquid surface interface) during the lysis step (Figure S9). DJ mixing is used hereafter during the magnetic depelleting procedure and is discussed further in the magnetic bead homogenization section. A total of 5 min of lysis mixing was ample for complete cell lysis, equating to 20 total DJ mixing iterations.

**Fig. 8:**
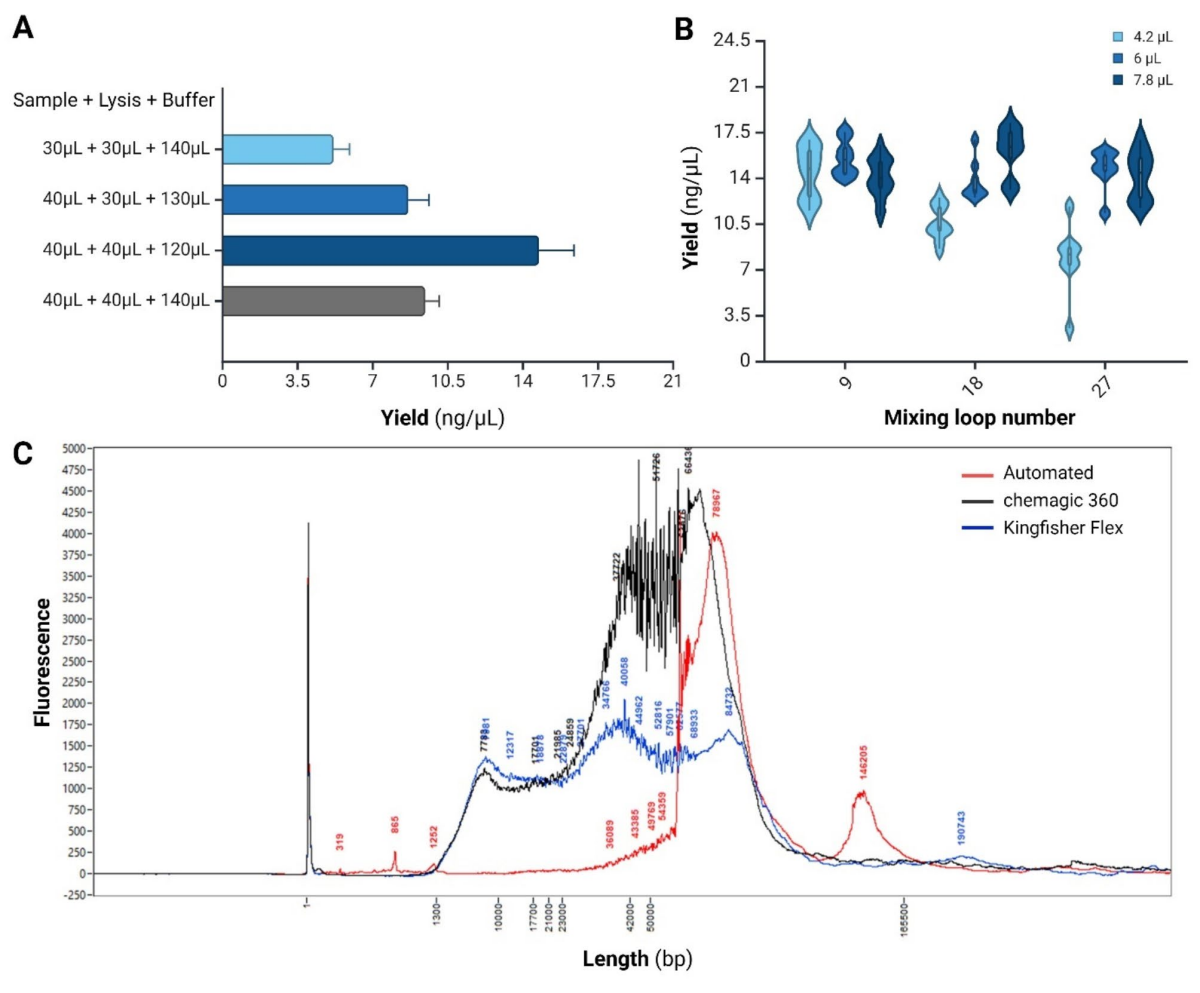
Comparison of NA recovery efficiency under different conditions in the automation system. (**A**) dsDNA yield (ng/µL) in different sample input volumes, lysis buffer, and binding buffer. (**B**) Violin distribution visualization of the recovered dsDNA concentration yield (ng/µL) and purity (260 nm / 280 nm) in different iterations of the bead volume (4.2 µL, 6 µL, and 7.8 µL) and the number of DJ mix loops (9, 18, and 27) during the washing step (*n*=8). (**C**) Electropherogram comparison of extracted dsDNA size distribution in length (bp) using three different automated DNA extraction methods: capillary-based automated extraction system (red line), chemagic 360 (black line), and Kingfisher Flex (blue line)

**Fig. 9:**
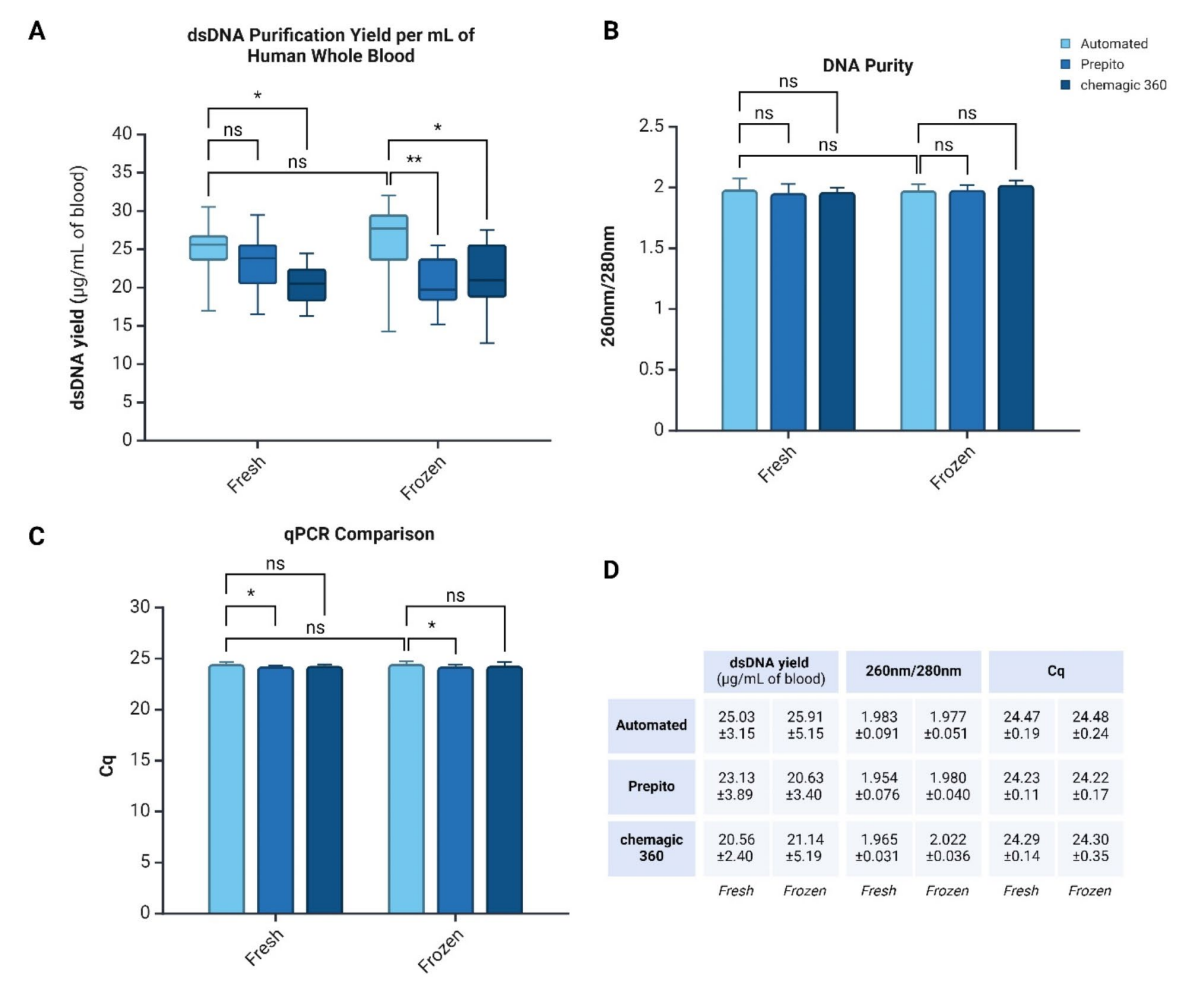
Comparison of the quantity and purity of extracted dsDNA between the automated capillary system and other automated NA purification workflows (Prepito and chemagic 360 instruments) from citrate-stabilized human peripheral blood in *Fresh* and *Frozen* conditions. (**A**) Purified dsDNA yield per 1 mL of human peripheral blood. (**B**) Assessment of DNA purity by absorbance ratio at 260 nm / 280 nm wavelengths. (**C**) DNA purity assessment by qPCR comparison targeting the reference Human ALB gene. Significant differences are indicated by ‘*’ *p* ≤ 0.05, ‘**’ *p* ≤ 0.01, and ‘ns’ P > 0.05 as no statistical difference (*n = 16 per condition and per workflow*)

Once lysed, NAs bind to the magnetic nanoparticles (MNPs) to isolate them from unwanted products [48]. Miniaturization of volume in a capillary system required adjusting the lysis buffer, binding buffer, and MNP ratio while maximizing the sample input volume (Fig. 3C **steps 3&4**). The highest yield of purified dsDNA (14.75 ng/µL) in the automation system was the most optimum at 1:1:3 for the input sample, the lysis buffer, and the binding buffer, respectively (Fig. 8A). Increasing the volume of sample input inherently increased recoverable dsDNA; concurrently increasing the volume of lysis buffer increased the efficiency of cell disruption, leading to a higher yield, as observed in automated workflows with increased sample input and lysis buffer at 40 µL. Given the maximum capacity of 200 µL of the plate well, the binding buffer needed to be decreased as the sample or lysis buffer increased. When the binding buffer exceeds this maximum capacity, homogenization becomes ineffective, and with the safe volume capacity, the recovered yield decreases, as seen in Fig. 8A. Although this guided the composition of the lysis behavior, the dsDNA yield was still comparable to other automation methods (Fig. 9). The bead binding capacity was optimized to improve the extraction method’s efficiency further.

After lysis, the mixture is incubated with MNP to bind NA before the MNPs are pelleted again and the supernatant is removed (Fig. 3C step 5). A safe aspiration height of 600 μm (equivalent to 5 µL leftover volume) between the tip of the cannula and the bottom of the 384-well plate resulted in the least bead disturbance. The distance between the tip of the cannula and the bottom of the 384-well plate was linearly correlated with the volume left in the well (Figure S11). It is essential to avoid aspirating any MNPs during supernatant removal, given that MNPs are crowded with the target NA. Subsequently, the cartridge cannula is washed with Tween-20 mix to remove any residue of supernatant waste (Fig. 3C step 6). Without cartridge washing, the purity of the NA (accessed by 260 nm/280nm wavelength absorbance) decreases, further compromising downstream processing (Figure S12).

After adding MNPs to the binding mix, strict mixing is required to resuspend the bead pellets to fully bind NA during washing and elution (Fig. 3C **steps 7–16**). The number of mixing loops and the amount of bead volume were adjusted to determine the highest and purest dsDNA recovery yield (Fig. 8B). DJ mixing is used here to depellet the MNPs by *Fast Mixing*, which allows beads to depellet by shearing forces induced by the collision of the bead with the well surface and the inside of the cannula tips. However, during *Fast Mixing*, the de-pelleted beads are susceptible to occupying only the bottom portion of the well. *Cyclic Mixing* is used to overturn the beads to the top area of the well to resuspend them better (Fig. 12C1). In fewer DJ mixing iterations (9 loops), the average recovered yield was relatively high (> 13 ng/µL) across different bead volumes (4.2 µL, 6 µL, and 7.8 µL); however, the purity of the samples was not consistent between all samples, 260 nm/280nm at 2.11 ± 1.21, 2.49 ± 0.58, and 2.05 ± 0.19 for each of the bead volumes, respectively, suggesting that the byproducts were not thoroughly washed. Intrinsically, as the amount of MNPs increases, the number of mixing loops needs to increase, but excess mixing potentially disengages the NA from the MNPs. Using a higher number of DJ mixing iterations (27 loops), 4.2 µL beads yielded 7.82 ± 2.59 ng/µL of NA; this is the lowest compared to all other conditions due to the NA being washed off during mixing before elution. The highest DNA concentration recovered (16.06 ± 1.88 ng/µL) with the most consistent purity, 260 nm/280nm of 1.86 ± 0.06, was in 7.8 µL beads with 18 DJ mixing loops. The increase in MNPs provided a larger surface area to which NA could bind, where 18 loops of DJ mixing removed most of the impurities while preserving the NA for library preparation. Additional iterations of increasing the mixing loop to homogenize the lysis buffer and sample (30 loops in 5 min), displacing the lysed sample into MNPs before displacing binding buffer to resuspend any leftover lysed sample, and washing the cartridge with Tween-20 further increased the overall dsDNA recovery yield to 21.12 ± 2.53 ng/µL (*n*=16).

The DNA fragments’ size distribution of the extracted dsDNA from blood using different automated extraction techniques was evaluated by the fluorescence intensity (Fig. 8C). Here, the chemagic 360 system showed a broad range of fragment sizes with a diverse distribution of DNA length, ranging from 2,000 bp to 130,000 bp dsDNA. In contrast, the Kingfisher Flex automated nucleic acid extraction system showed a consistent distribution of fragment sizes but with a lower intensity of large dsDNA, where fragments above 20,000 bp did not show an intensity of more than 2,000 relative fluorescence units (RFU). On the other hand, the capillary-based automation system preserved the long sequence of gDNA from 30,000 bp to 150,000 bp dsDNA. The distribution of the fragments using different automation methods is most likely influenced by the mechanical shearing processes during sample handling with magnetic beads. For example, the Kingfisher Flex system uses repetitive oscillatory up and down motion by the rod when handling samples to homogenize solution, and the gDNA likely experiences increased shear force. The chemagic 360 system uses rotational motion by the rod to homogenize the solution and likely decrease the experienced shear force. The Kingfisher Flex and the chemagic 360 system liquid handling method utilize magnetic rods to displace MNPs instead of the liquid. In contrast, the capillary-based extraction method displaces the supernatant and avoids displacing MNPs; here, the combination of *fast mixing* and *cyclic mixing* preserves a larger distribution of dsDNA.

Having optimized the entire extraction and purification process, our platform was tested with 32 citrate-stabilized human peripheral blood samples stored at 4 °C (*Fresh*) and − 20 °C (*Frozen*) conditions (16 samples per condition). The input samples were purified using the presented automation system and other automated extraction workflows, such as the Prepito and chemagic 360 nucleic acid extraction instrument, to assess the recovery efficiency and quality of library prep-ready dsDNA. Here, the capillary-based automation workflow used 40 µL of peripheral blood input eluted in 33 µL of elution buffer. In comparison, the other automated workflows (Prepito and chemagic 360) used the standard operation protocol of 400 µL of peripheral blood input eluted in 200 µL of elution buffer. The effectiveness of the extraction was evaluated by comparing the recovered dsDNA yield of 1 mL of input peripheral blood and the recovered dsDNA quality for each workflow and storage condition (Fig. 9A). There were no significant differences in the dsDNA yield for *Fresh* and *Frozen* conditions using automated workflow. Similarly, in the *Fresh* condition, the automated workflow had no significant differences from Prepito workflow; however, the automated workflow had a higher median dsDNA yield compared to chemagic 360 with statistical significance (*p ≤ 0.05). For the *Frozen* condition, the median dsDNA yield was higher in Prepito (** *p* ≤ 0.01) and chemagic 360 (*p ≤ 0.05) (Fig. 9D). Continuous stringent mixing with precision liquid handling is expected to be the reason for the higher average yield. There were no significant differences in purity in all workflows and conditions (Fig. 9A), indicating a relatively efficient purification process for all workflows.

Given that the input volume and the elution volume ratio differed for each of the workflows compared (82.5% for the presented automation system, 50% for the other automated workflows), the final eluted dsDNA concentration was also different – *Fresh* condition: Automated workflow 30.34 ± 3.82 ng/µL, Prepito workflow 46.26 ± 7.79 ng/µL, chemagic 360 workflow 41.13 ng/µL; *Frozen* condition: Automated workflow 31.41 ± 4.79 ng/µL, Prepito workflow 41.26 ± 6.80 ng/µL, chemagic 360 workflow 42.29 ± 10.38 ng/µL. The eluted dsDNA was subsequently added to a qPCR reaction targeting the housekeeping reference albumin (ALB) gene to further evaluate the quality of the recovered dsDNA, comparing Cq values. Here, the automated workflows had no significant difference between *Fresh* and *Frozen* conditions and chemagic 360 workflows; however, a difference was observed compared to Prepito (*p ≤ 0.05). As expected, this was due to the lower total input dsDNA mass (given the fixed input volume) added to the qPCR reaction in the automated workflow than other automated instruments (Fig. 9C).

The sequencing library was prepared manually using the DNA extracted from the mentioned workflows and conditions to evaluate the potential capacity of the sequencing qualities when automated. Observed with an electropherogram, a high-quality and comparable sequencing library was generated (Figure S13). The CV percentages of recovered dsDNA yield in all conditions showed < 30%, while the CV percentages in the 260 nm / 280 nm ratios showed ≤ 5%, and the Cq value showed ≤ 1% (Figure S14). The quality and quantity of extracted dsDNA provided enough evidence to incorporate into automated library preparation.

### Optimization of library preparation steps – post-NAP

The optimization principles mentioned above, preconditioning, thermal control of the fluid slug, and movement of the fluid slug, were used in the library preparation steps: fragmentation, adenylation, ligation, and PCR. However, three additional automation challenges are addressed in this section: (1) pressurization of the flexible tubing, (2) contamination of the tube surface with endonuclease, and (3) management of viscous ligation and fragmentation buffers.

The next step after gDNA extraction is the fragmentation of purified gDNA, which is the initial setup of library preparation (Fig. 3C steps 17, 18 and Fig. 3D step 1). This process involves endonuclease, a vital component of the fragmentation mix. Endonucleases simultaneously break both strands of the dsDNA while generating nicks on each strand, resulting in a normalized distribution of sheared DNA [14]. The fragmentation activity of gDNA is maximized at 35 °C, and nucleases denature at 65 °C under capillary pressure. It is worth noting that fragmentation is still active at room temperature but is marginal; hence, controlling the fragmentation rate with the correct position of the slug relative to the heating zone, controlled temperature, and well-regulated duration yields the desired standard fragments. During any thermal treatment, it is imperative to pressurize the volume slugs in the heating zone for two reasons: (1) to provide a closed system preventing any volume loss due to evaporation and (2) to improve thermal conductivity from heating block to flexible tubing by expanding the tubing, creating more intact surface contact. The localized pressure in the heating zone is created by a triangular valve with a low surface area that pinches the flexible tubing before the air is pumped by the syringes (Figure S4 A-C). The pinch valves could hold over 50 PSI pressure in all 16 tubes (8 tubes per cannula).

**Fig. 10:**
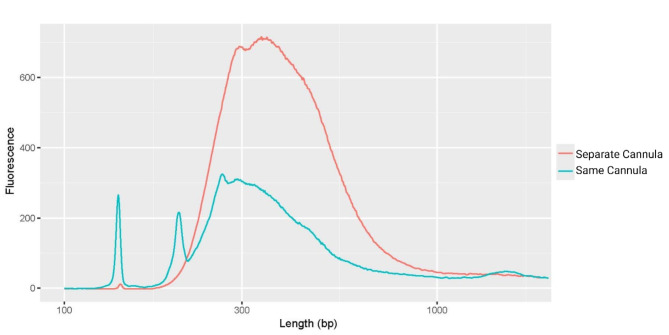
Electropherogram evaluation of sequencing ready library when using separate (red) and same (blue) cannula during the fragmentation step. The separate cannula uses a Y-junction design of the consumable cartridge to move fluid unidirectionally

As the fragmentation mixture is aspirated towards the heating element, some residual restriction endonucleases can adhere to the inner tube walls between the cannula tips and the heating zone (Figure S2A). These leftover traces can negatively impact subsequent steps since restriction endonucleases are inactivated at high temperatures, typically around 65 °C [49, 50]. However, the area between the cannula tips and the heating zone never reaches this temperature. Incorporation of restriction endonuclease contaminants from the tubing poses the danger of chewing up any barcode indexes and further fragmenting the DNA, ultimately reversing the ligation of adapters to the fragmented DNA and further cleaving the library within the polynucleotide chain. The downstream activity of the nuclease contamination yields an unsequenceable library with high adapter dimers, as observed within the libraries prepared using the same cannula tubing instead of a new surface area (Fig. 10). Therefore, the Y-junction of the cartridge design allows liquid to move in a single direction, where the fluid contacts only the uncontaminated tubing area, and the treated fragmentation mixture is dispensed into the ligation mixture using a separate cannula (Figure S4).

**Fig. 11:**
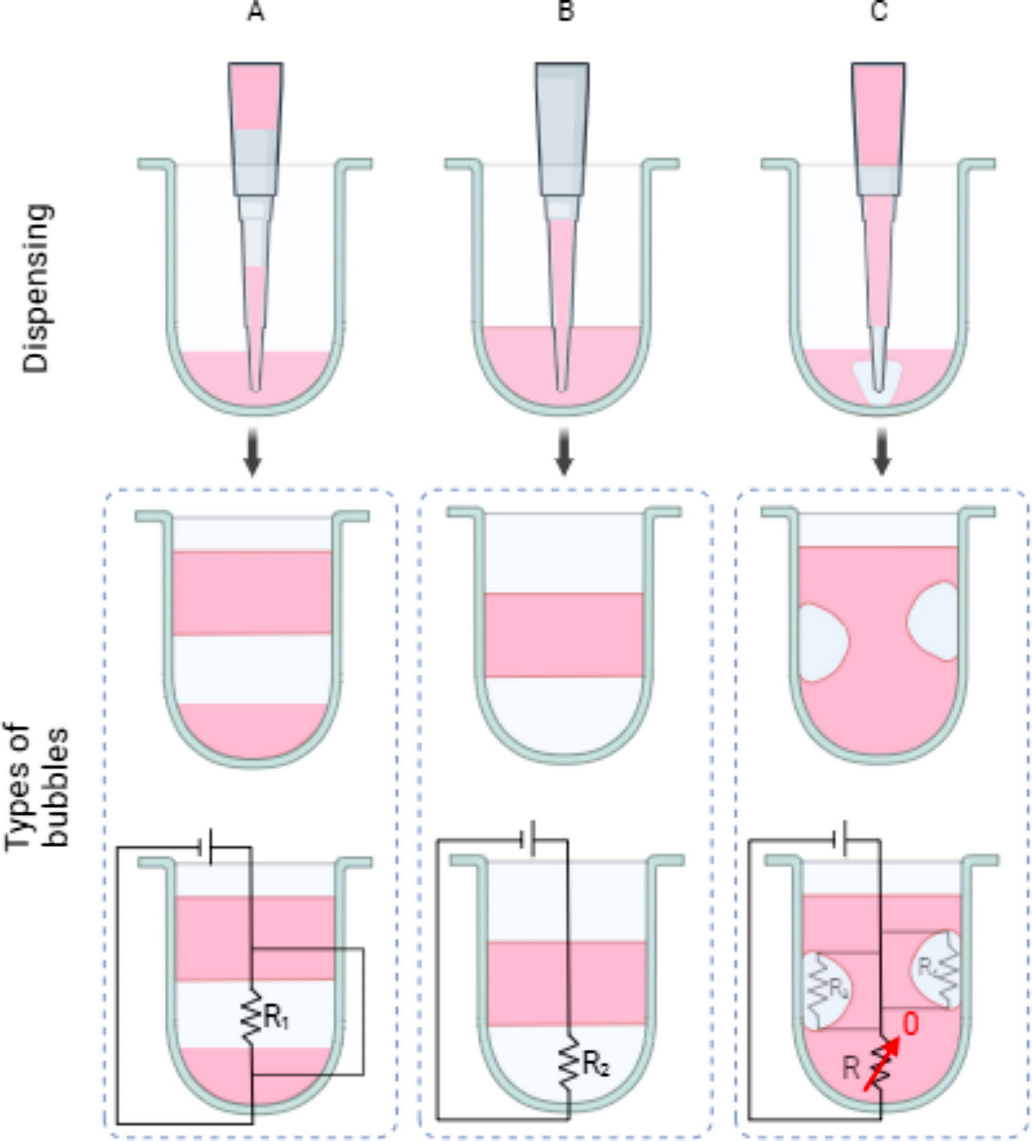
Types of air bubble formation during liquid dissolution into a narrow deep-well plate, analogous to an electrical circuit with resistors. (**A**) An air bubble formed between the liquid segments. All the liquid can be collected during aspiration, but bubbles will not be removed, leading to uneven location of the slug in the heating zone. (**B**) An air bubble formed at the bottom of the well of the plate, the most common form of bubbling. (**C**) Air bubble pockets formed on the side of the plate well where there is no direct effect on the aspiration volume. However, they will inherently increase the theoretical liquid height, affecting further downstream aspiration and blowout procedures

Complete homogeneous mixing of the buffer, enzyme mix, and purified input gDNA is again crucial for complete normalized fragmentation. Here, the inherent viscous property of the fragmentation and ligation mixture imposes bubble formation during liquid handling and mixing. Three different types of bubble formation were observed for automation systems working with narrow deep-well plates (200 µL, 384-well plates), all detrimental to capillary-based heating. The continuity of fluid slugs can be understood similarly to an electrical circuit where bubbles are analogous to resistors (Fig. 11). A bubble formed between two segments of fluid slug (Type A; Fig. 11) and a bubble formed at the bottom of the well (Type B; Fig. 11**)** affect the position of the fluid after aspirating to the heating zone, comparable to increased resistance in a circuit. Such bubbles form and remain intact when large volumes of air are introduced during dispensing.

**Fig. 12:**
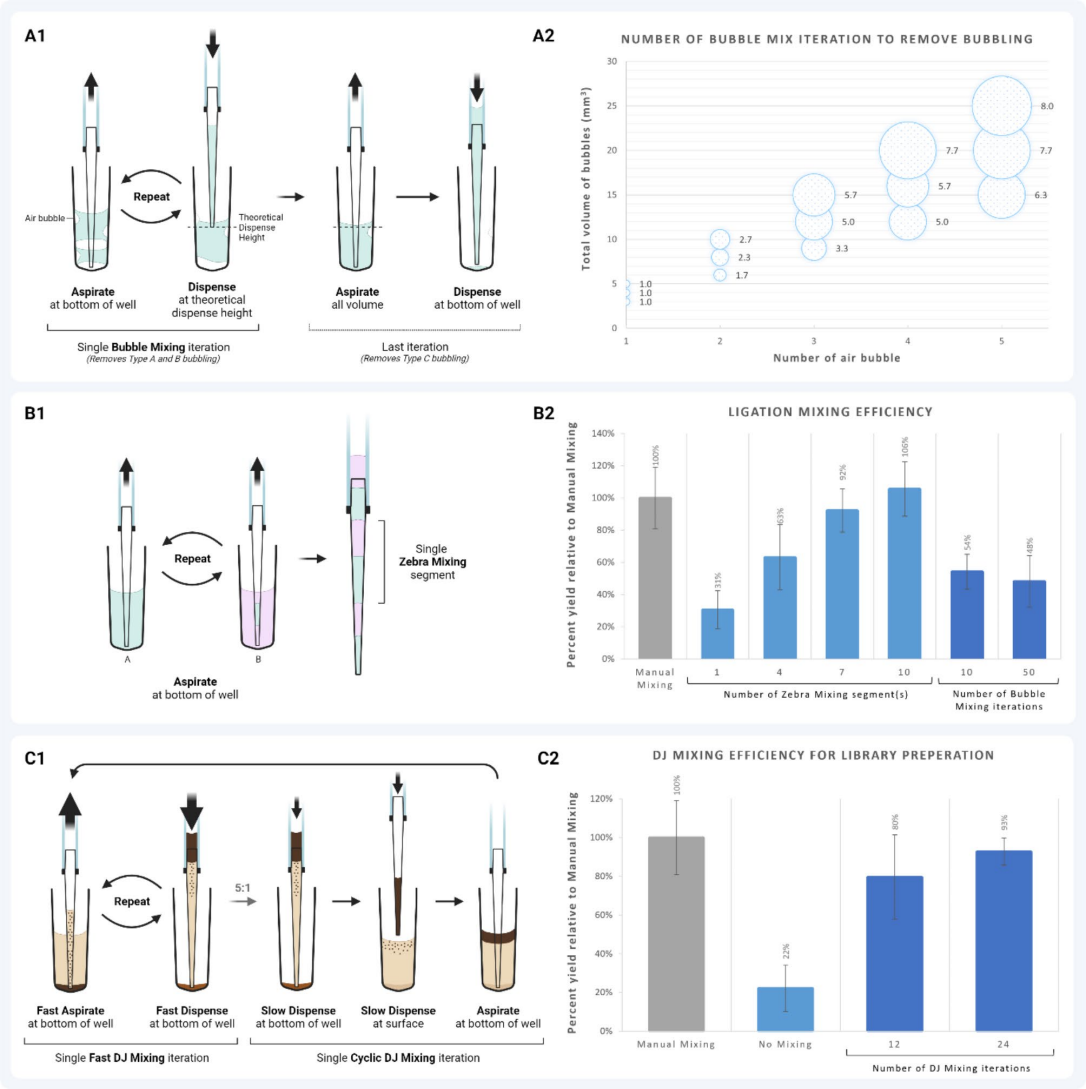
The different mixing techniques used for library preparation. (**A1**) Bubble mixing method to burst air bubbles formed during dispense. In a single bubble mixing iteration, the pipette tip is moved to the bottom of the plate well and aspirated equivalent to 35% of the total mixed volume to dispense at the theoretical dispense height (the predicted volume height when no air bubble is present), removing all Type A and Type B bubbling. The mixed volume is aspirated at the bottom of the plate well and dispensed back to remove any Type C bubbling. (**A2**) The average number of bubble mixing iterations required to remove a set number of air bubbles at different volume capacities (*n*=3). (**B1**) Zebra mixing method for homogenizing up to two highly viscous reagents. A single Zebra Mixing segment consists of equal portions of the two reagents, each of which is aspirated sequentially from the bottom of the plate well. (**B2**) Comparison of manual mixing efficiency to Zebra and bubble mixing efficiency to produce sequencing-ready libraries, estimated by the percent library yield compared to manual bench-top mixing method. (**C1**) DJ Mixing method for stringently resuspending magnetic bead particles during the NA purification process. The entire cycle consists of 5 *Fast* mixing iterations (aspirating from the bottom of the plate well then jet dispensing at high speed) for every 1 *Cyclic* mixing iteration (dispensing 75% of the total aspirated volume at the bottom of the plate well before dispensing the remaining volume above the liquid surface interface). (**C2**) Library preparation efficiency using 12 and 24 DJ mixing iterations compared to Manual and No Mixing methods by the percent yield of the sequencing ready library

To remove any formed bubbling, a bubble mixing technique (Fig. 12A1) is used, which is the iterative aspiration of air or liquid at the bottom of the well and dispensing at the theoretical volume height (the predicted height of the liquid and air interface when no bubbles are present). This will eradicate the two types (A and B) of bubbling. However, the third type of bubbling (Type C; Fig. 11) will not be eradicated here because a bubble, similar to an established closed-circuit system with passive resistors, does not stress the continuity of the slugs. This bubbling occurs when a small volume of air is introduced to the slugs, which is not large enough to disrupt the continuity but adheres to the side of the well and affects the theoretical volume height. Increased volume height has adverse effects during downstream blowout dispensation, which is a fast dispensation method above the surface of the fluid to exude any remaining liquid at the end of the cannula tips. A modified bubble mixing technique with a subsequent aspiration of the total liquid volume in the well and dispensing after iterative bubble mixing removes this bubble type (Type C; Fig. 11). At least 8 iterations of bubble mixing at an aspirating capacity of 35% of the total liquid volume will remove the worst-case scenario of the highest total air volume of air at an average of $\:25\:m{m}^{3}$ in 5 individual air bubble capsids (Fig. 12A2).

After removing bubble formation, the fragmented and end-repaired NAs were homogenized with the ligation mix (Fig. 3D step 2). The sticky end of the fragmented dsDNA has an overhang of adenine, which ligates with the overhang of the adapter barcodes with the help of ligase. The high viscosity of the ligation mix is due to the high molecular weight buffer and the concentration of ligase, making mixing the treated fragmentation mix and the subsequent ligation and adapter mix challenging. Conventional mixing of aspiration and dispensing at high speed does not result in a homogeneous mix due to the strong intramolecular force. It is also susceptible to the formation of irreversible tiny clusters of bubbles. The Zebra mixing technique was created to minimize the formation of tiny bubbles and homogenize vicious components. Iteratively aspirating small portions of the two reagents creates minimally distanced layers of differentiable viscous liquid, which creates a zebra pattern of two fluids. The volume of the fluid layer should be minimized at 6 µL bounded at 10% of the total mixed volume per aspiration. It is worth noting that the mixed volumes should be approximately equal before Zebra mixing to produce the most uniform mixing. At least 10 Zebra mix segments are needed to homogenize the ligation mix (Fig. 12B2). Once the fragments are adapter ligated, the polished DNA undergoes magnetic bead-based purification similar to the pre-library preparation NAP.

To selectively preserve the polished fragments and wash away any unwanted adapter dimers (a by-product of two adapters binding to one another), a molecular size-selective ratio of bead volume and IPA is used for washing steps (Fig. 3D steps 3–12). It is important not to disturb the bead pellets while washing beads with IPA, as resuspended beads can elute DNA from their surface; therefore, a safe dispensing and aspirating height of 400 μm from the bottom of the well to the tip of the cannula is used during bead washing. This was equivalent to ~ 4 µL of IPA (~ 10–15% of the final volume), where increased IPA leftover volume can lead to a decrease in the recovery of dsDNA (Figure S15). The leftover IPA is dried at room temperature before being eluted.

The polished purified fragments are further amplified using capillary-based PCR cycles similar to the heating steps previously mentioned in pretreatment (Fig. 3D step 14). Overshooting and undershooting the desired temperature are essential metrics for efficient amplification, and the PID feedback system is extensively used here. Again, during PCR cycles, the amplified fluid slugs must be pressurized and placed well relative to the heating zone. Finally, the amplified products use the same purification procedure as the pre-amplification procedure but with beads at the same volume ratio as the post-PCR product to maximize the elution of amplified DNA (Fig. 3D steps 15–23). In between the washing and heating steps, the cartridge tubing is washed repeatedly with tween-20 and isopropyl mixture to remove any residual material, as shown in Fig. 3C & D. To prevent any splash contamination or extended evaporation of reagents from the 384-well plate, a prepierced 384-well seal was adhered to the top of the plate during the NAP and Library preparation processes.

### Prepared library sequencing

**Fig. 13:**
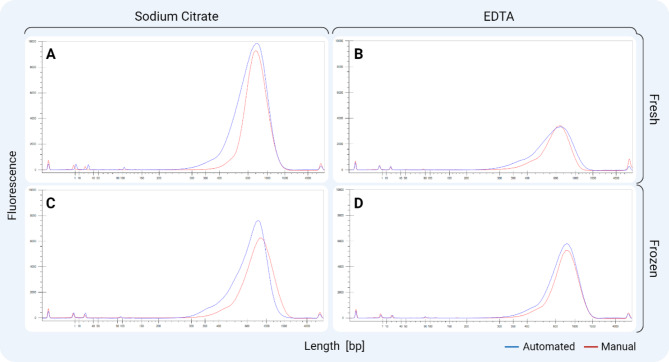
Overlaid comparison of the size distribution of libraries obtained from automated (blue) and manual (red) NAP and library preparation workflows.(**A**) Citrate-stabilized *Fresh*-storage sample, (**B**) EDTA-stabilized *Fresh*-storage sample, (**C**) Citrate-stabilized *Frozen*-storage sample, and (**D**) EDTA-stabilized frozen-storage sample. The visual representation allows for evaluating potential differences in the electropherogram profiles resulting from the different library preparation methods and sample storage conditions

A final volume of 35 µL purified library ready for NGS was constructed using automated and manual workflows from citrate and EDTA-stabilized human peripheral blood under storage conditions of 4 °C and − 20 °C. The mass yield of the generated library and the molecular weight size distribution were consistent in both workflows. A normal library distribution was observed under all conditions, ensuring uniform sequencing coverage. This is particularly important for accurate variant detection and quantification to cover the area of interest while avoiding sequencing biases. The average size of the library peaked at ~ 700 bp, where the fully automated and manual purification and library preparation protocol showed a similar distribution and concentration of the library (Fig. 13). No adapter dimers (size 120–170 bp) were exhibited, showing efficient purification of the library post-adapter ligation and post-PCR steps. The sequenced libraries’ concentrations ranged between 200 and 2000 bp size across different conditions were compared. The peripheral blood samples kept with citrate stabilization exhibited a higher library concentration in both *Fresh* and *Frozen* storage conditions compared to samples obtained in EDTA collection tubes, respectively – $\:48.34\frac{ng}{\mu\:L}$ and $\:31.78\frac{ng}{\mu\:L}$ compared to$\:\:17.98\frac{ng}{\mu\:L}$ and $\:20.30\frac{ng}{\mu\:L}$ for automated workflow and $\:22.16\frac{ng}{\mu\:L}$ and $\:22.08\frac{ng}{\mu\:L}$ compared to$\:\:10.74\frac{ng}{\mu\:L}$ and $\:17.46\frac{ng}{\mu\:L}$ for manual workflow (Figure S16). This may be due to calcium chelation in EDTA blood samples, which artificially activates platelets during collection and can release the enclosed DNA, further degrading potential sequence-able material before library preparation. (51–52) The library concentration yield between *Fresh* and *Frozen* samples was similar in each anticoagulated condition, providing flexibility in different types of input samples for the automation system.

**Fig. 14:**
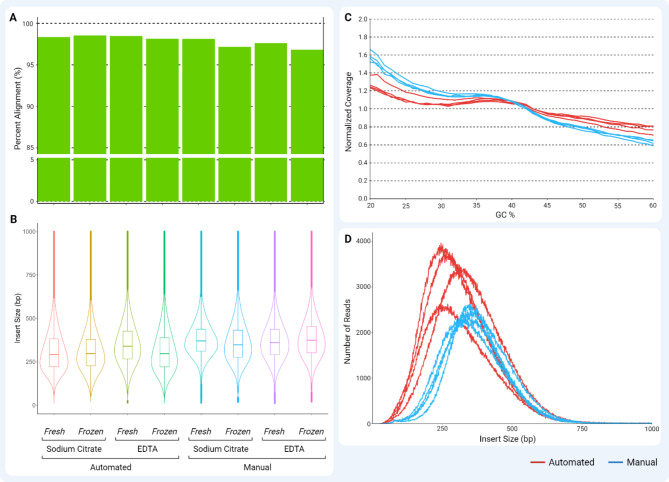
Comparison of sequencing data from libraries prepared in automated (red) and manual (blue) workflows. Citrate-stabilized and EDTA-stabilized Human peripheral blood stored in *Fresh* and *Frozen* conditions are compared in (**A**) Percent alignment to the Human Genome assembly GRCh38, (**B**) Insert size distribution visualized in the violin distribution graph with minimum, Q1, median, mean, Q3, and maximum sizes, (**C**) normalized coverage with GC bias seen in 20–60% of the GC content representing 95% of the human genome, and (**D**) Number of reads compared to insert size grouped by workflow

The efficiency of adapter ligation and amplification quality is often reflected in the quality of sequencing data metrics, including base quality, insert length, percent alignment, percent dimer, GC bias in normalized coverage, and the total number of reads. The quality scores of the different samples were comparable for automated and manual workflows. Sequencing samples were aligned with Human Genome assembly GRCh38, where the automated workflow had samples preserved in citrate stabilization with a percentage alignment of 98.34% and 98.53%, and samples preserved in EDTA with a percentage alignment of 98.47% and 98.15% stored in *Fresh* and *Frozen* conditions, respectively [53]. This was comparable to a manual workflow where samples preserved in citrate stabilized samples had percentage alignment of 98.12% and 97.17%, and samples preserved in EDTA had percentage alignment of 97.62% and 96.82% stored in *Fresh* and *Frozen* conditions (Fig. 14A). Furthermore, the automated workflow had an average insert size of 313.5 bp and 313.5 bp for citrate-stabilized samples and 357.4 bp and 317.5 bp for EDTA-stabilized samples stored in *Fresh* and *Frozen* conditions, respectively. Compared to manual workflow, the insert size had a similar average of 387.0 bp and 368.5 bp for citrate-stabilized samples and 387.0 bp and 390.0 bp for EDTA-stabilized samples stored in *Fresh* and *Frozen* conditions (Fig. 14B). As expected, adapter-dimer sequences were not observed under all conditions. A similar normalized coverage of 20–60% GC bias was observed between all samples, representing 95% of the human genome library, which did not exhibit biases in sequence content (Fig. 14C). As expected, the shorter insert sizes in the automated workflow had more reads, compensating for more coverage for the template genome without compromising alignment (Fig. 14D). Overall, the quality and quantity of the sequencing and libraries prepared using automated workflow was comparable to manual workflow.

## Discussion

The presented study demonstrated the development and implementation of a novel automated system for preparing an NGS-ready DNA library directly from peripheral blood samples. Implementing automated liquid handling systems and simplified protocols improved reproducibility, with no pipette changing procedure, and reduced hands-on time, addressing major challenges in the NGS library preparation workflow. By minimizing human error and standardizing pipetting steps, the automated system produced high-quality extracted nucleic acid comparable to other automated liquid handling systems and consistent sequencing capacity. This is particularly important for scaling genomic studies and clinical applications where reproducibility is critical. The reduction in hands-on time and overall turnaround time achieved through automation and one-step workflows is a key advantage of the system for low-throughput laboratories seeking to increase efficiency and bypass any manual sample preparation.

The capillary-based automation system presented unique challenges that are particular to the flexible tubing used for the workflow. Without using a drop-tip mechanism, the system utilized a single disposable cartridge system and a 384-well plate. Preconditioning of the flexible tubing provided the adaptability of the capillary tubing to be used in enzyme-dependent thermal reactions, while the Y-junction design of the tubing situated the cartridge to avoid cross-contamination issues. The combination of viscosity fixed slug velocity, cannula tip mixing techniques, and pressurized capillary heating allowed a distinct method of automating liquid displacement, homogenization, and thermal treatment for purification and amplification steps.

However, the system has several limitations that should be addressed in the future. One of the primary limitations of the automated system is the initial plate loading process, which is susceptible to pipetting errors, particularly given the small size of the 384-well plate. This issue could be mitigated by preloading the reagents onto the plate before distribution to users, which can be available to users directly as a complete kit. Alternatively, a small-scale displacement automation system could be utilized to preload the necessary reagents onto the 384-well plate before the user loads samples. Another limitation is the inability to quantify purified and extracted NA before proceeding with library preparation. In clinical research, DNA yield can vary based on the cell count in blood samples, necessitating normalization before library preparation [54]. This issue could be addressed by using samples with similar white blood cell counts or modifying the MNPs used in the nucleic acid extraction method to normalize the purified NA with an upper threshold [55–58]. Additionally, using sequencers with a higher total number of reads (for deeper sequencing depth) in future analysis may provide a more complex comparison of sample types and the effect of the automation system on the quality of the final libraries, such as the leaching of pretreatment solution or loss of genetic material.

The automated system’s ability to handle both fresh and frozen blood samples stabilized with either citrate or EDTA highlights its versatility without compromising the integrity of the sequencing data. Compared to other existing automated liquid handling systems, capillary-based automation presents a small benchtop footprint with minimum consumables and a low barrier of entry. Given the low-throughput capacity of the system, the automation system is highly suitable for laboratories of mid-sized and smaller sizes with limited technical expertise, adding to the efforts to streamline the NGS workflow for decentralized settings.

## Conclusions

This study showcased the transformative potential of the miniaturization of an automation system to streamline the critical steps for the extraction of gDNA and preparation of the NGS library from EDTA and citrate stabilized human peripheral blood stored in *Fresh* (4 °C) and *Frozen* conditions (-20 °C). The automation design offers full liquid handling, precise temperature control, efficient heat transfer, and minimized fluid loss for the nucleic acid purification and library preparation workflow, ensuring consistent yield at a low-throughput scale. Furthermore, through novel cartridge designs and mixing techniques, capillary tubing systems addressed specific challenges in library prep, such as fragmentation, ligation mixing, bubble formation, and contamination. The platform generated high-quality NGS libraries with minimal user intervention at the start of the run, which was demonstrated by the quality of the sequencing results comparable to that of manual workflows. Furthermore, the system required minimal consumables – an off-the-shelf well plate and a custom-built cartridge, making it ideal for labs of all sizes and facilitating the widening of the adoption of NGS in decentralized settings. In general, these advances pave the way for more accessible, efficient, reliable, and standardized NGS workflows, useful across various research fields.

## Electronic supplementary material

Below is the link to the electronic supplementary material.

Supplementary Material 1

## Data Availability

The datasets used in this study for comparison are available in the NCBI library Genome assembly GRCh38 (NCBI RefSeq assembly: GCF_000001405.26).

## Abbreviations

NGS: Next–Generation Sequencing
SBS: Sequencing by synthesis
WGS: Whole genome sequencing
NA: Nucleic acid
NAP: Nucleic acid purification
PCR: Polymer chain reaction
PoC: Point of care
PNK: Polynucleotide kinase
PEG: Polyethylene glycol
SPRI: Solid phase reversible immobilization
IPA: Isopropyl alcohol
TPE: Thermoplastic elastomer
BSA: Bovine serum albumin
ddNTPs: Dideoxynucleotides
dsDNA: Double–stranded DNA
gDNA: Genomic DNA
DJ: Depelleting jet mixing
MNP: Magnetic nanoparticle
ALB: Albumin
