# Supplementary material for: Peripheral blood to next-generation sequencing ready DNA library: a novel engineering design for automation
Source: BMC Genomics. 2024 Oct 22;25:987. doi: 10.1186/s12864-024-10892-0 (PMC11494769; doi:10.1186/s12864-024-10892-0)
Supplement: Supplementary file 1 — Supplementary Material 1 [file 12864_2024_10892_MOESM1_ESM.pdf]

Supporting Information

Peripheral Blood to Next-Generation Sequencing Ready DNA Library: A Novel Engineering Design for Automation

Dulguunnaran Naranbat[1], Lothar à Brassard[2], Nabil Lawandy[1], and Anubhav Tripathi[1*]

[1]Center for Biomedical Engineering, School of Engineering, Brown University, Providence, RI 02912, USA

[2]Revvity chemagen Technologie GmbH, Arnold-Sommerfeld-Ring 2, Baesweiler, 52499, Germany

[*To whom correspondence may be addressed. E-mail: anubhav_tripathi@brown.edu]

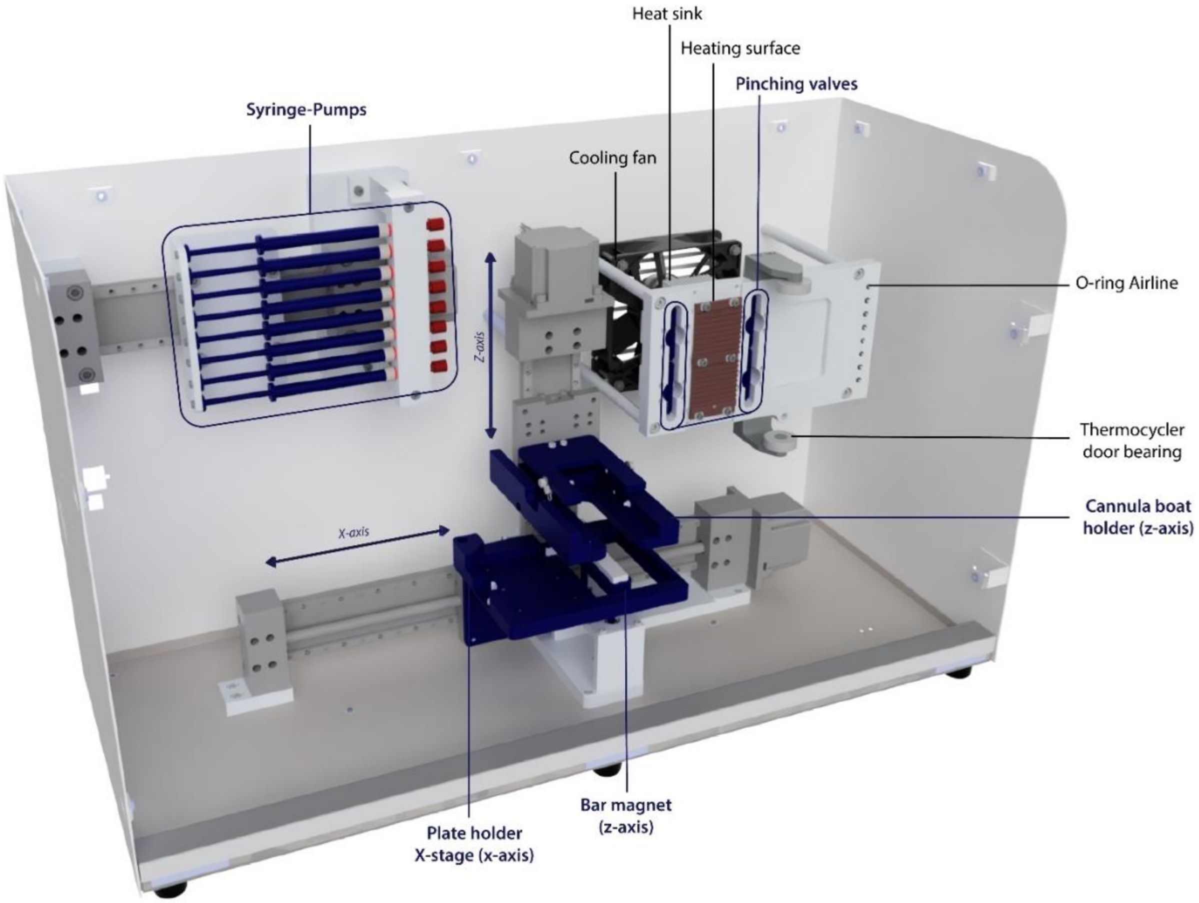

Figure S1. Device assembly and active components for the automation process.

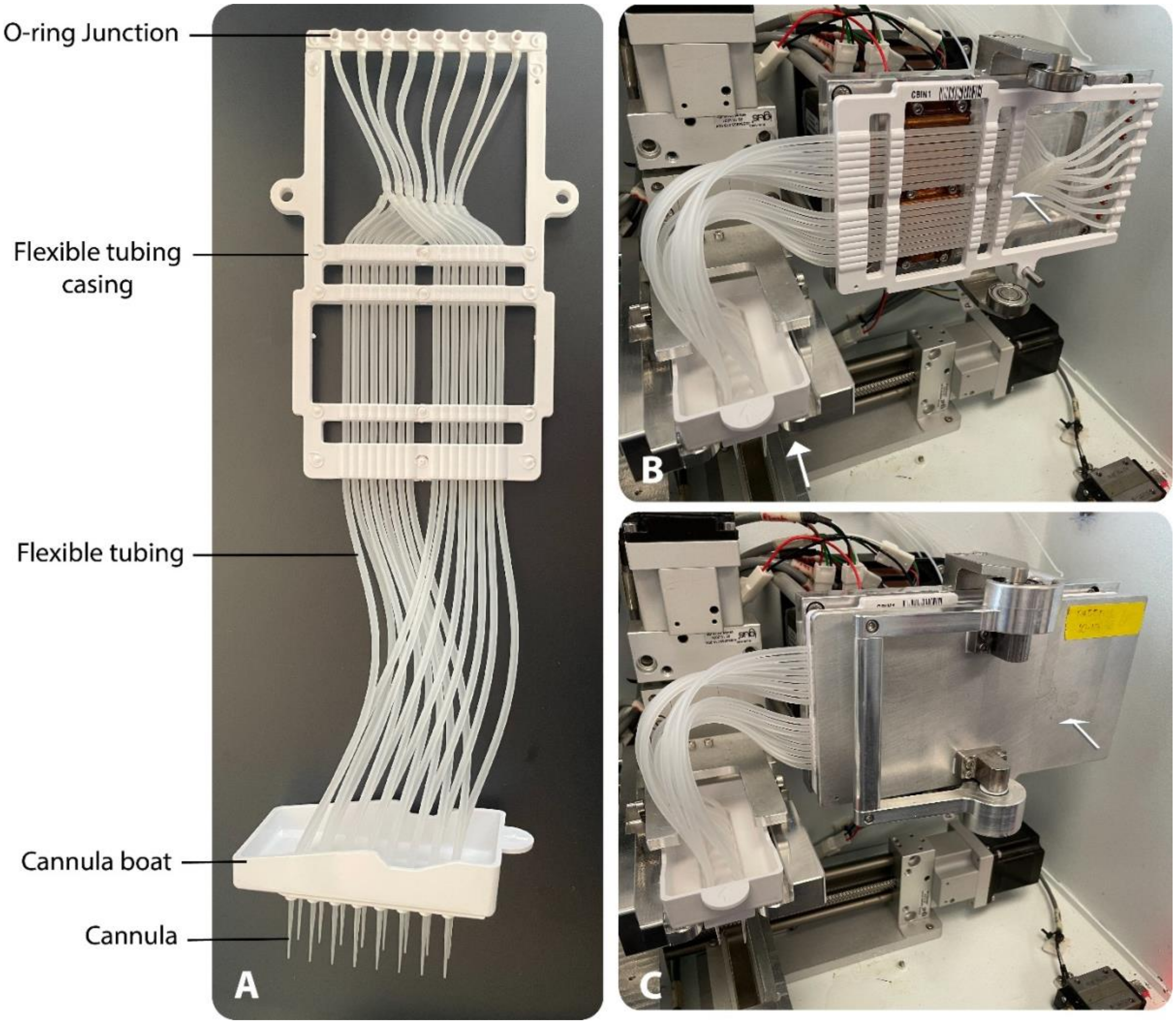

Figure S2. Consumable capillary tubing cartridge. **A**) single-use tubing consists of 16 individual Flexible tubing attached to Cannula tips held together by a Cannula boat and a flexible tubing casing. **B**) The flexible tubing is inserted into the automation device through two placeholders – the Cannula boat and the Flexible tubing casing. **C**) After placement, a detachable door with insulation padding is inserted in front of the casing to keep the tubing in place.

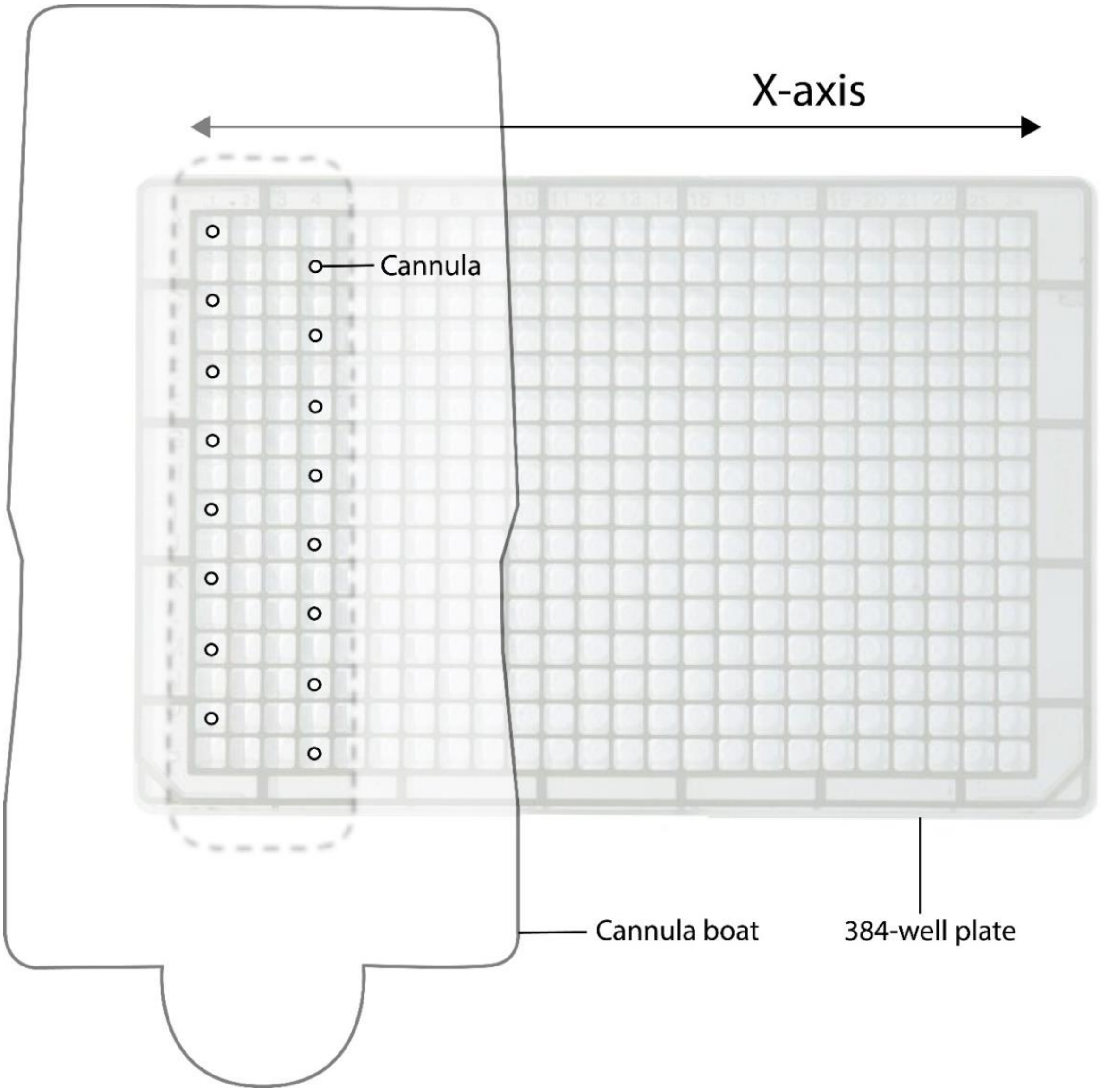

Figure S3. 384-well plate overlay with the Cannula boat. The Cannula tips are shown as black circles as a schematic of where each cannula tip is placed during the experimental run.

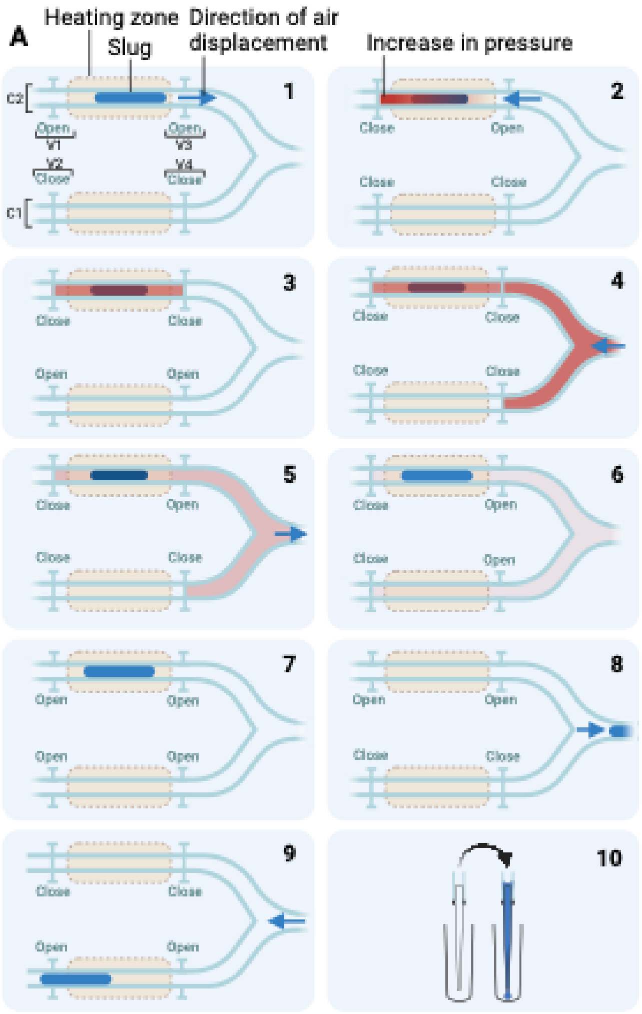

B

	Valve 1	Valve 3	Valve 2	Valve 4
1	Open	Open	Closed	Closed
2	Close	Open	Close	Close
3	Close	Close	Open	Open
4	Close	Close	Close	Close
5	Close	Open	Close	Close
6	Close	Open	Close	Open
7	Open	Open	Open	Open
8	Open	Open	Close	Close
9	Close	Close	Open	Open

Figure S4. Schematic view of the heat pressurization sequence and Y-junction slug motion. **A** – **1**) The consumable cartridge has two lanes (C1 and C2) joined together by a Y-junction. Cannula 2 (C2) has two valves (V1 and V3), while Cannula 1 (C1) has two valves (V2 and V4). The liquid slugs from the 384-well plate are displaced to the C2 heating zone (brown dashed rectangle) with air displaced by the syringe pump, where only C2 is open to atmospheric pressure – this is done by having V2 and V4 closed while V1 and V3 are open. Initially, the slugs are moved slightly to the right of the heating zone. **2**) To increase the internal tubing pressure around the liquid slug, V1 is closed, and the syringe pump increases the air and pressurizes the air around the slug (increased pressure shown by red gradient coloring inside the tubing). **3**) V3 is closed to keep the high pressure (red coloring) between V1 and V3 in C2 during heating. V2 and V4 are opened to let the non-pressurized zone (C1) reach atmospheric pressure. **4**) After the thermal treatment, the valves V2 and V4 are closed to create pressure in the Y-junction with the pressure pump. **5**) V3 is slowly opened to normalize the pressure in C2 and the Y-junction. Here, the syringe pump is expanded to decrease pressure in both zones. **6**) The area between V2 and V4 (C1) is at atmospheric pressure; therefore, V4 is slowly opened to normalize the pressure past V1 and V2 to be as close to atmospheric as possible. **7**) All the valves are opened where all areas are normalized to atmospheric pressure. **8**) The V2 and V4 are close to displacing the slug from C2 to the Y-Junction area. **9**) The valves are inversed – V1 and V3 closed (C1) while V2 and V4 are opened (C2) to move the slug through the Y-junction and past the other cannula (C1) for dispensing. **10**) Treated slug is dispensed into the selected well. **B** – The sequence of the valve positions (closed or open) to create internal pressure and displace liquid through the Y-junction.

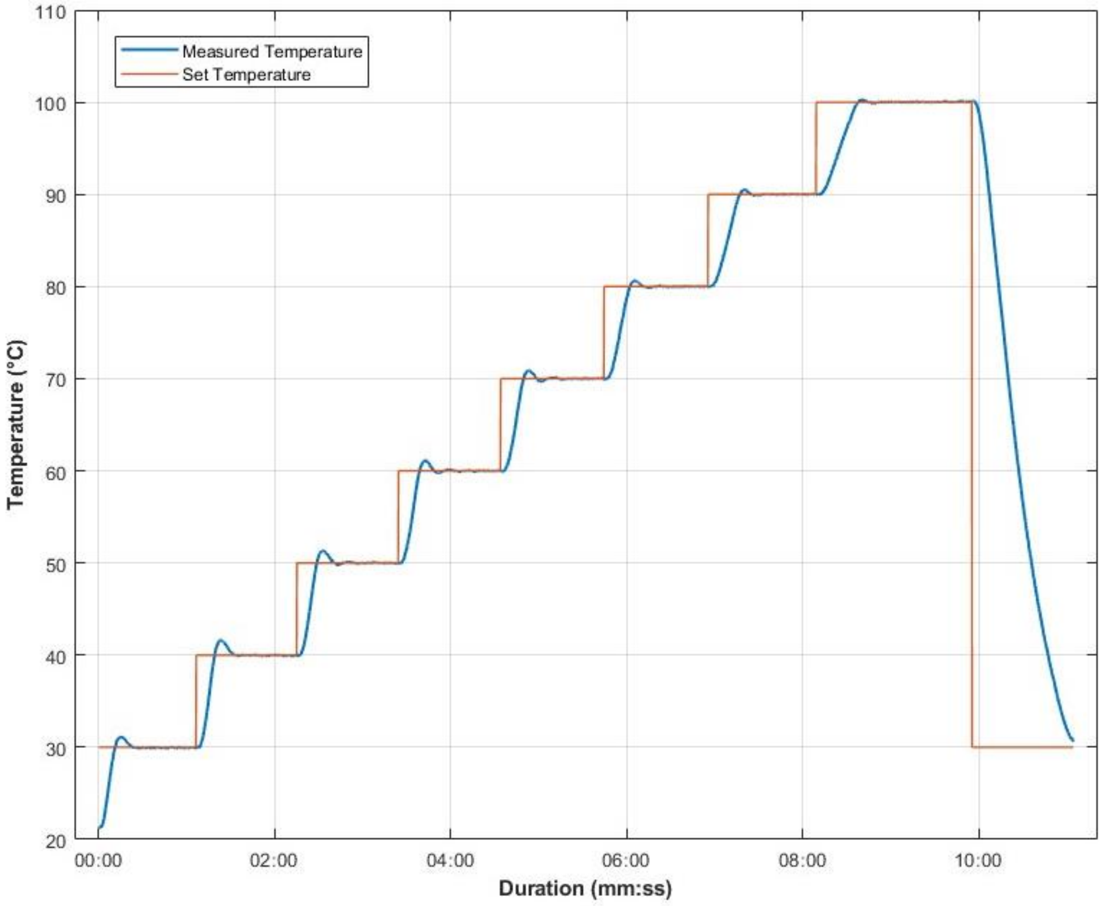

Figure S5. Temperature (20 - 100°C**)** profile of a PID-regulated heating system in the automation device with ~75 seconds duration in each step of temperature change (+10°C). The measured temperature (Blue line) from the thermocouple follows the heating system set temperature (Brown line) from the control console.

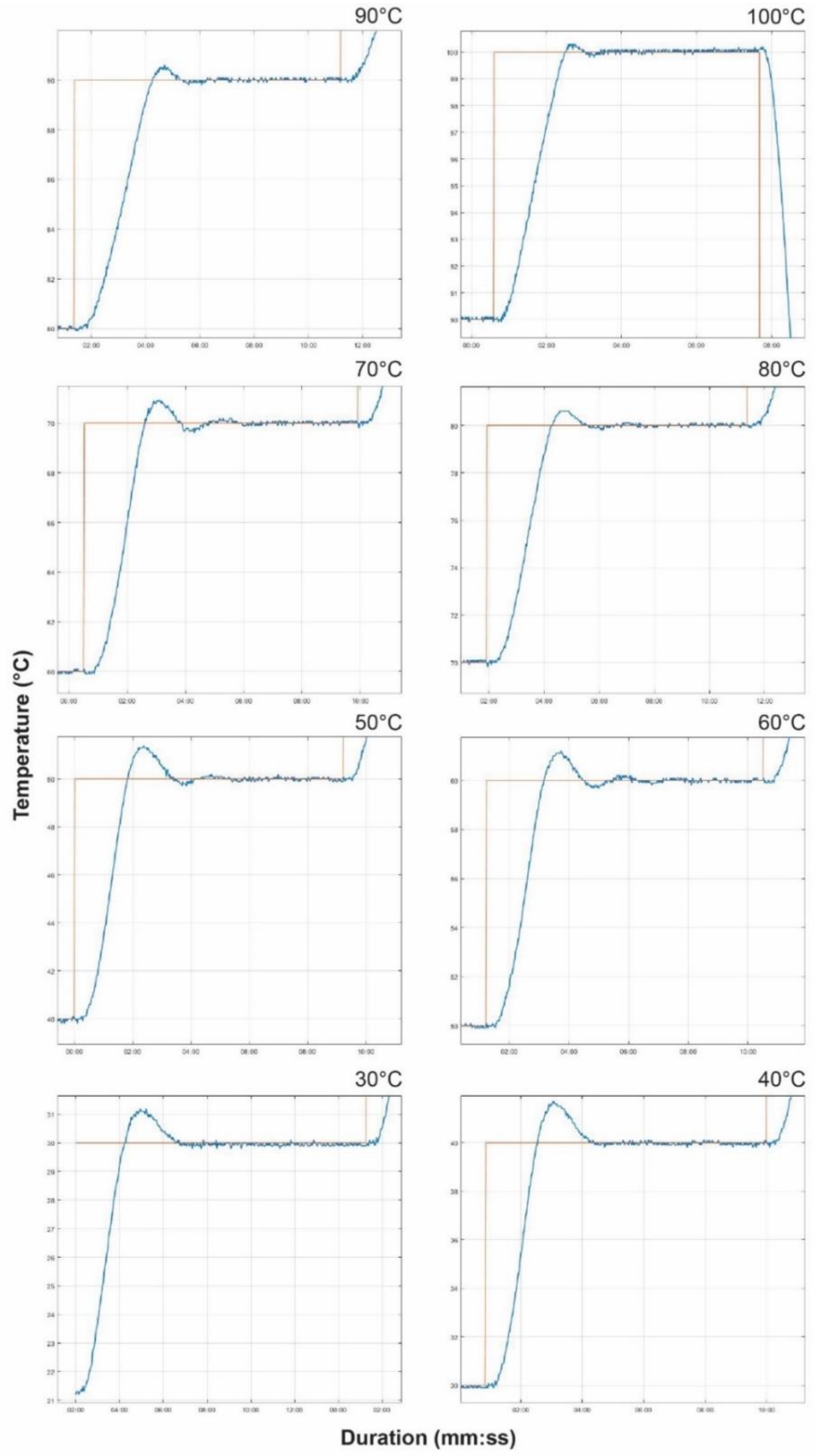

Figure S6. Temperature regulation profiles at each temperature zone have different ramp-up rates, cool-down rates, overshooting, and standard errors in maintaining the set temperature.

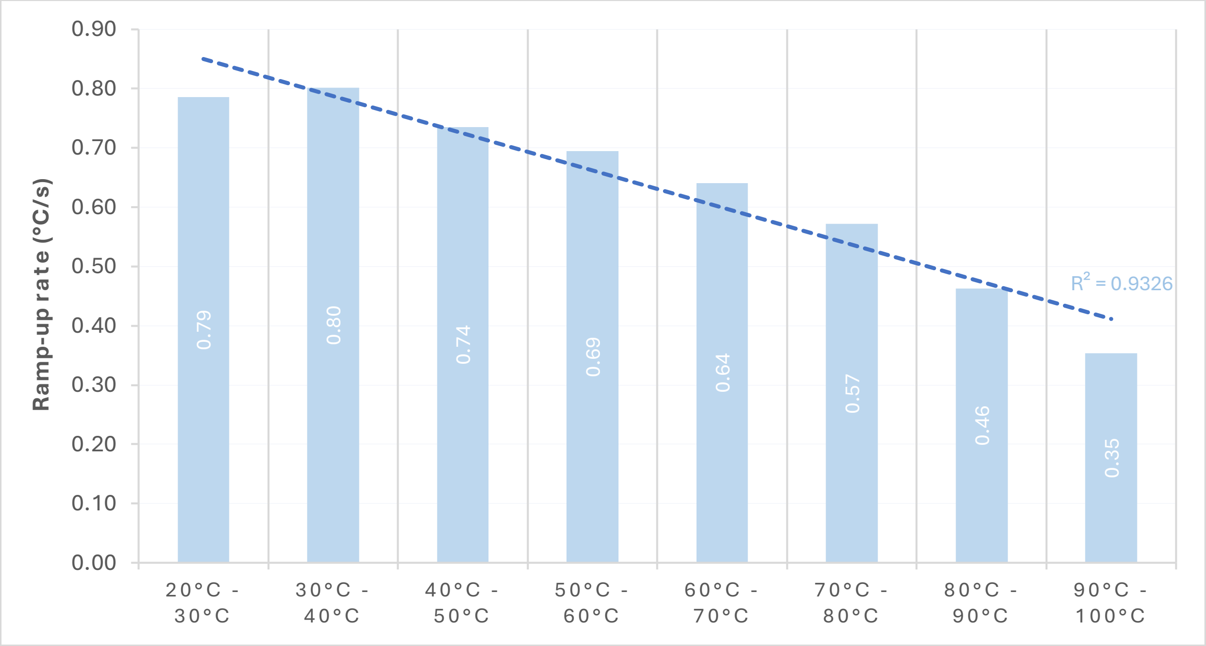

Figure S7. The ramp-up rate of the heating system is from when the heating command is registered until the inside of the flexible tubing reaches the desired temperature at different temperature zones.

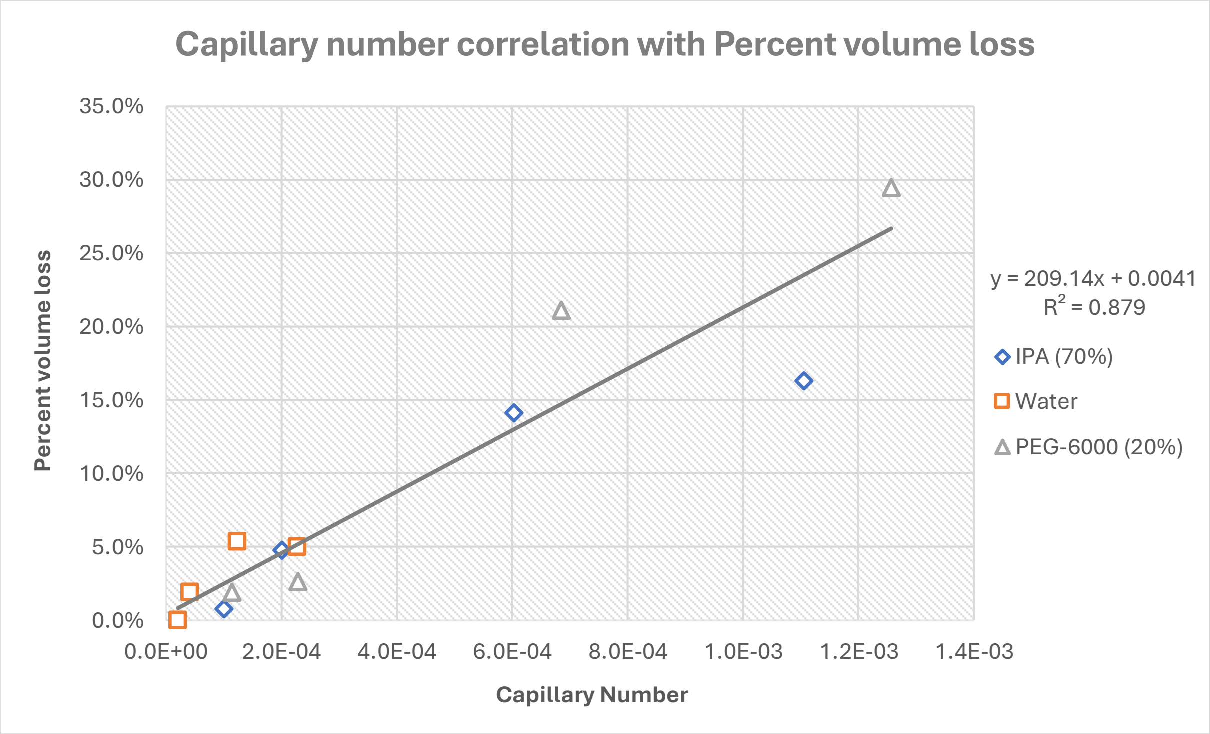

Figure S8. Correlation of capillary number with percent volume loss in IPA (70%), Water, and PEG 8000 (20%) during fluid motion in the flexible tubing liquid handling steps. The capillary number is dependent on the velocity of the fluid motion.

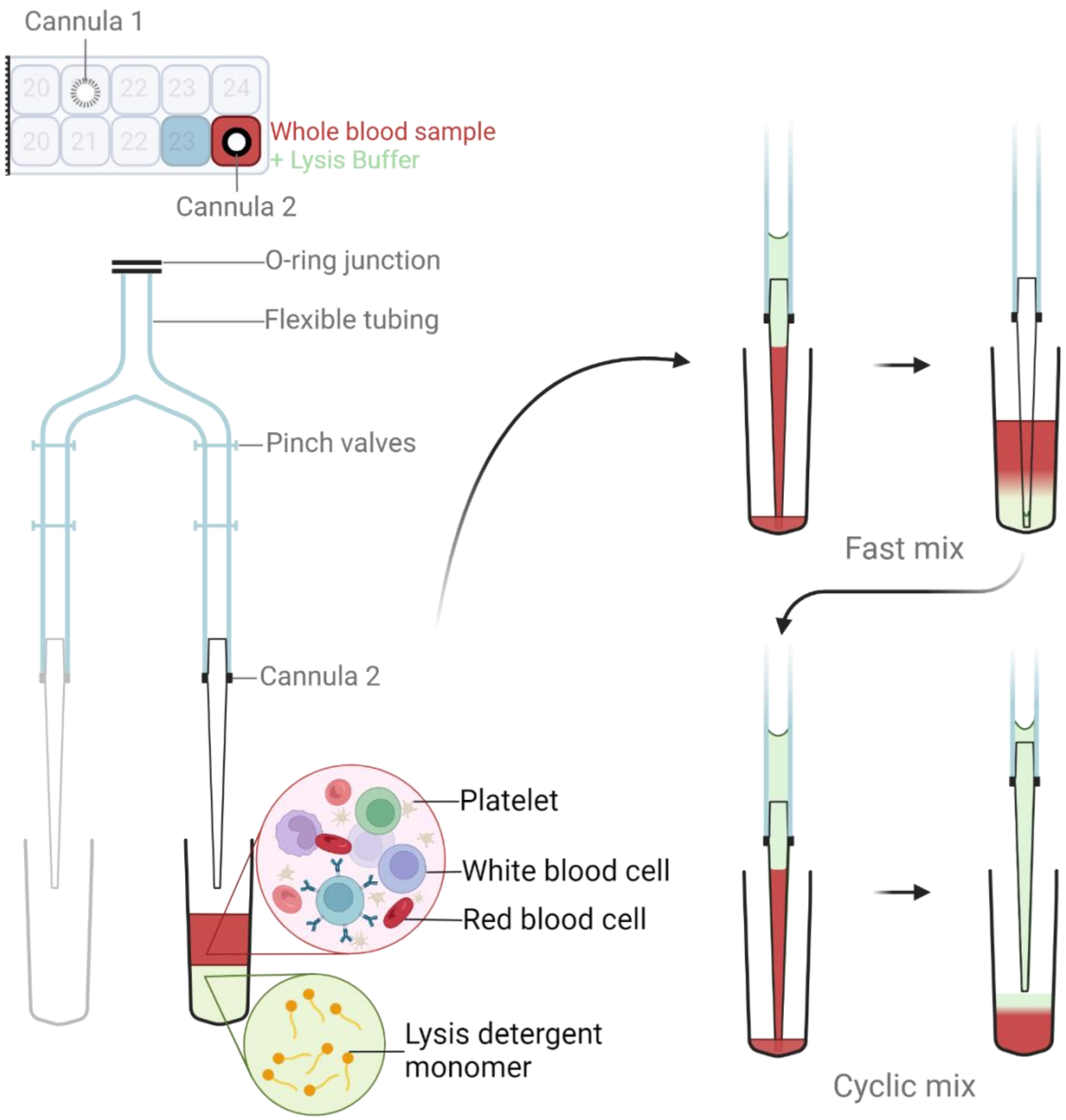

Figure S9. The lysis buffer, containing the detergent monomers, is mixed with the peripheral whole blood sample for complete white blood cell and nuclease membrane lysis using a stringent single cycle of De-pelleting Jet (DJ) mixing technique at a ratio of 5 *Fast mixes* for every 1 *Cyclic mix* iteration.

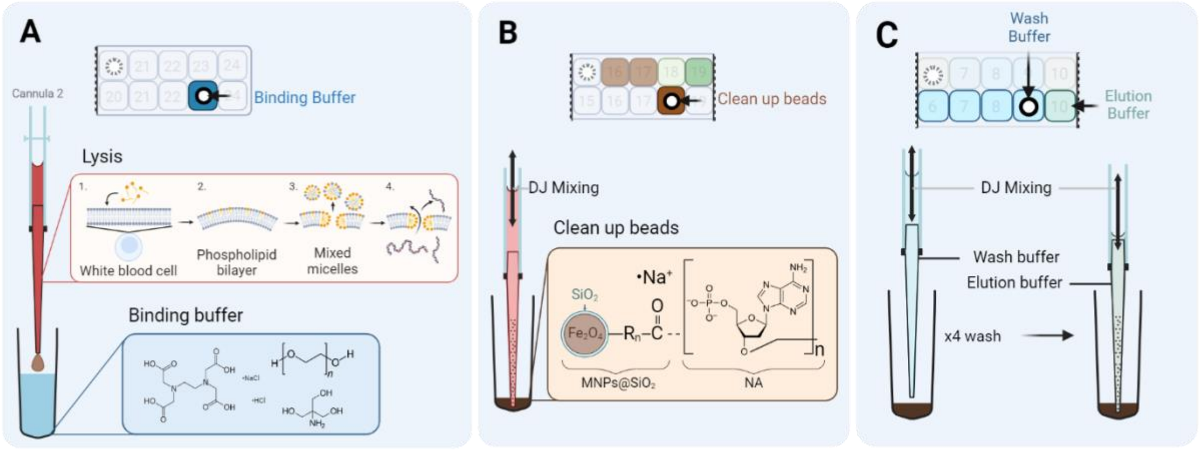

Figure S10. Schematic overview of cell lysis, NA binding, washing, and elution steps in the purification process. **A**) the lysis detergent monomers actively break down the phospholipid bilayer by creating mixed micelles, ultimately suspending the NA in a binding buffer solution, which incorporates binding enhancers such as NaCl, EDTA, Tris-HCl, and PEG. **B**) The lysed sample with the binding buffer is stringently mixed with MNPs to reversibly bind NA to the particles while washing away unwanted by-products, which are eventually eluted for library preparation. **C**) 4 repeats of bead washing to remove any unwanted byproducts from the lysis before eluting off all the bound NA from the MNPs using a water-based elution buffer.

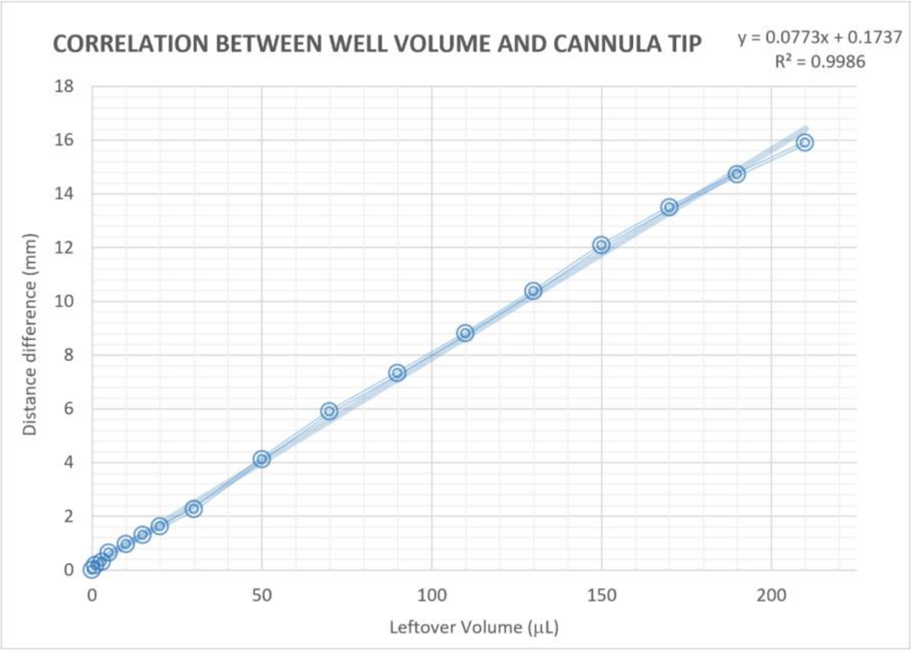

Figure S11. Correlation of leftover volume in the well and the distance between the cannula tip & 384-well plate bottom. Linear correlation between the two variables is observed with a gradual, well-shaped shape and round bottom.

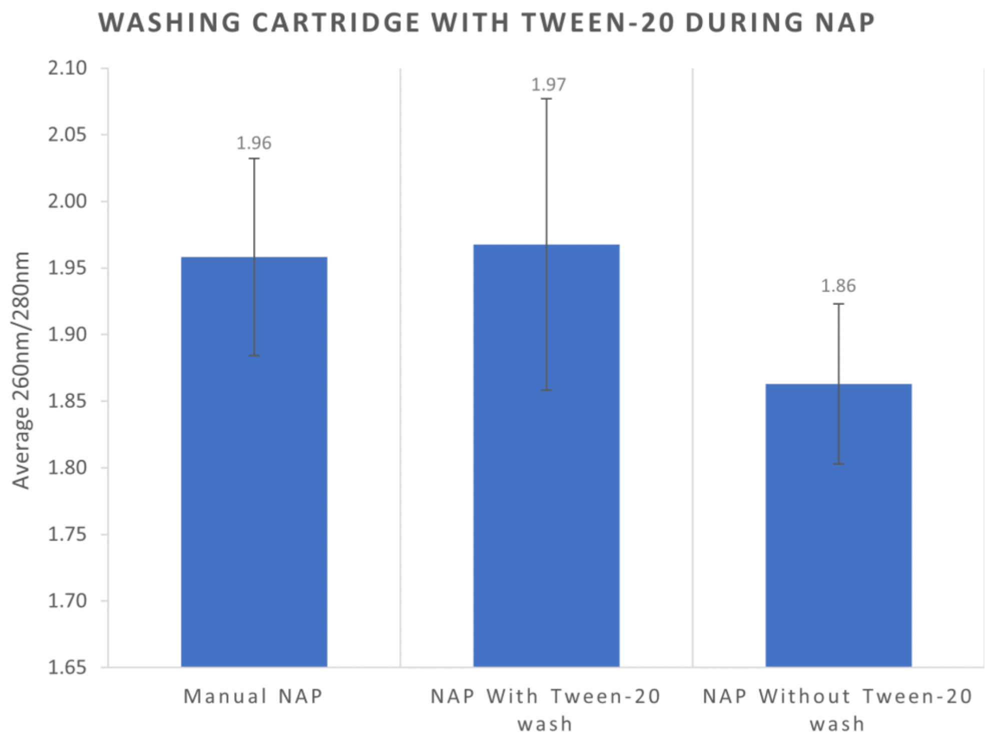

Figure S12. Effect of washing cartridge with Tween-20 during nucleic acid purification (NAP) procedure. Average 260nm/280nm ratio of the recovered NA in each condition to access the purity of the purified samples – Manual purification protocol, Purification with Tween-20 cartridge washing, and Purification without tween-20 cartridge washing.

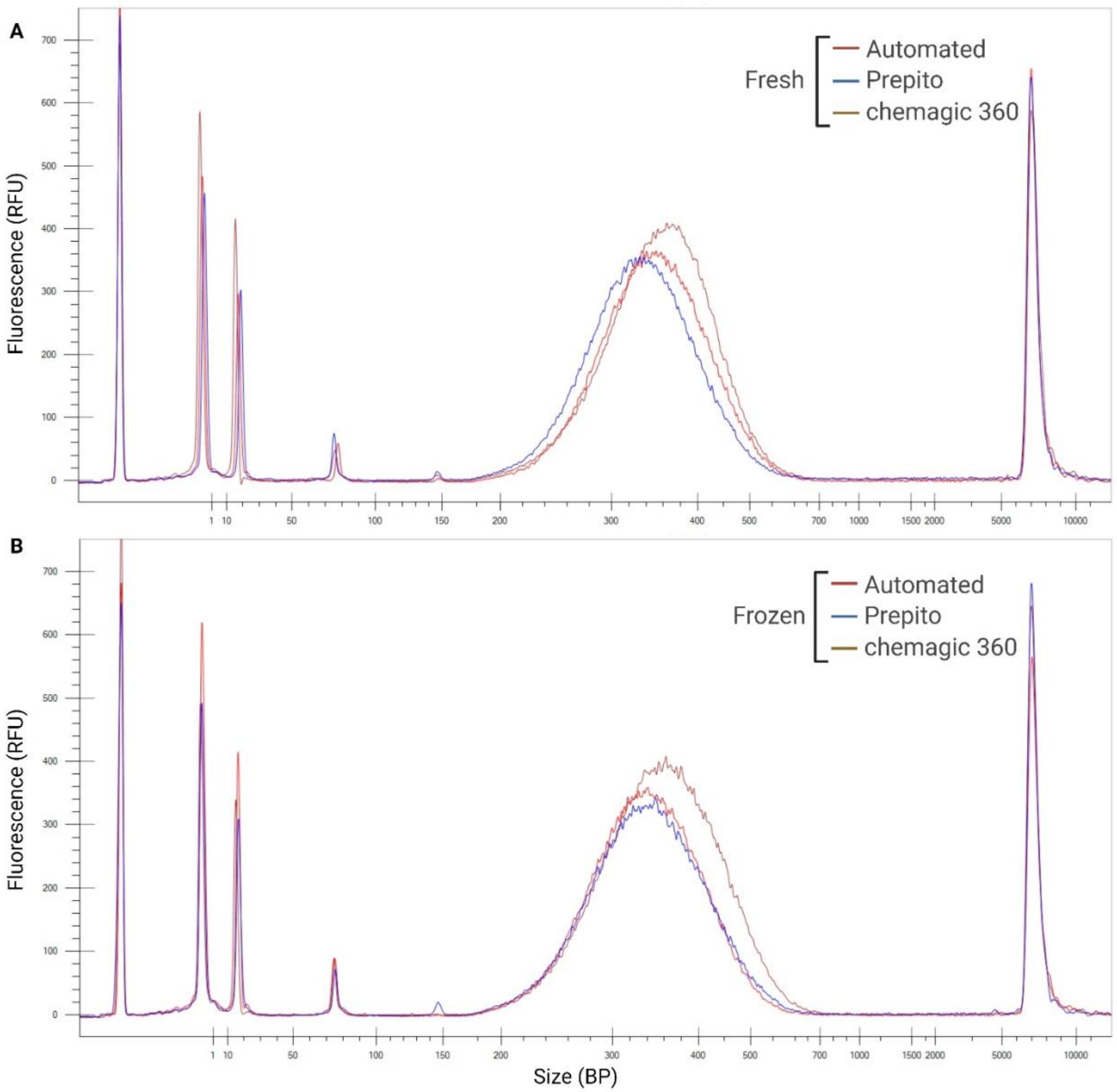

Figure S13. Overlayed size distribution analysis of sequencing-ready libraries. Libraries were prepared manually using NA extracted from Automated and other automated workflows (Prepito and chemagic 360 instruments)—compared peripheral blood samples stored in **A**) *Fresh* and **B**) *Frozen* conditions. The libraries exhibited similar fluorescence (intensity) and size distribution across all conditions.

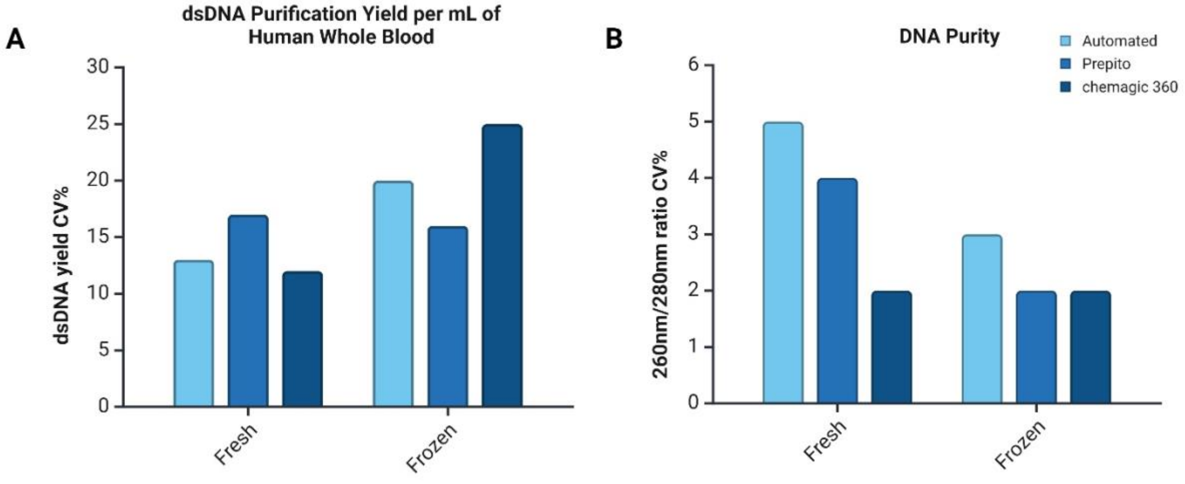

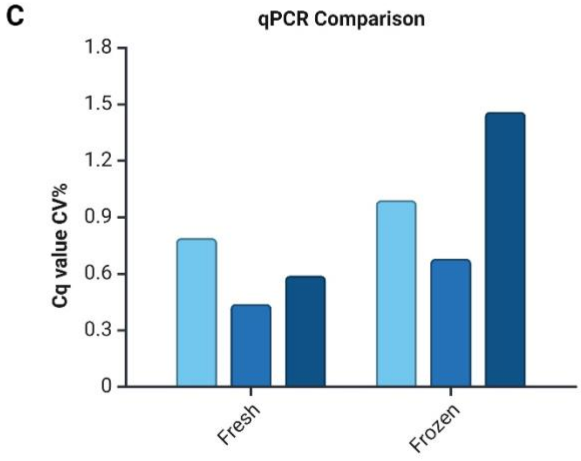

D

	dsDNA yield (µg/mL) CV %		260nm/280nm CV %		Cq CV %	
Automated	13%	20%	5%	3%	0.79%	0.99%
Prepito	17%	16%	4%	2%	0.44%	0.68%
chemagic 360	12%	25%	2%	2%	0.59%	1.46%
	Fresh	*Frozen*	*Fresh*	*Frozen*	*Fresh*	*Frozen*

Figure S14. Percent CV comparison of Automated and Manual NA purification protocols from samples stored in *Fresh* and *Frozen* conditions. **A**) CV% of purified dsDNA yield per 1 mL of Human peripheral whole blood. **B**) CV% of DNA purity assessed by absorbance ratio at 260nm/280nm wavelengths. **C**) CV% of DNA purity assessment by qPCR comparison targeting reference Human ALB gene.

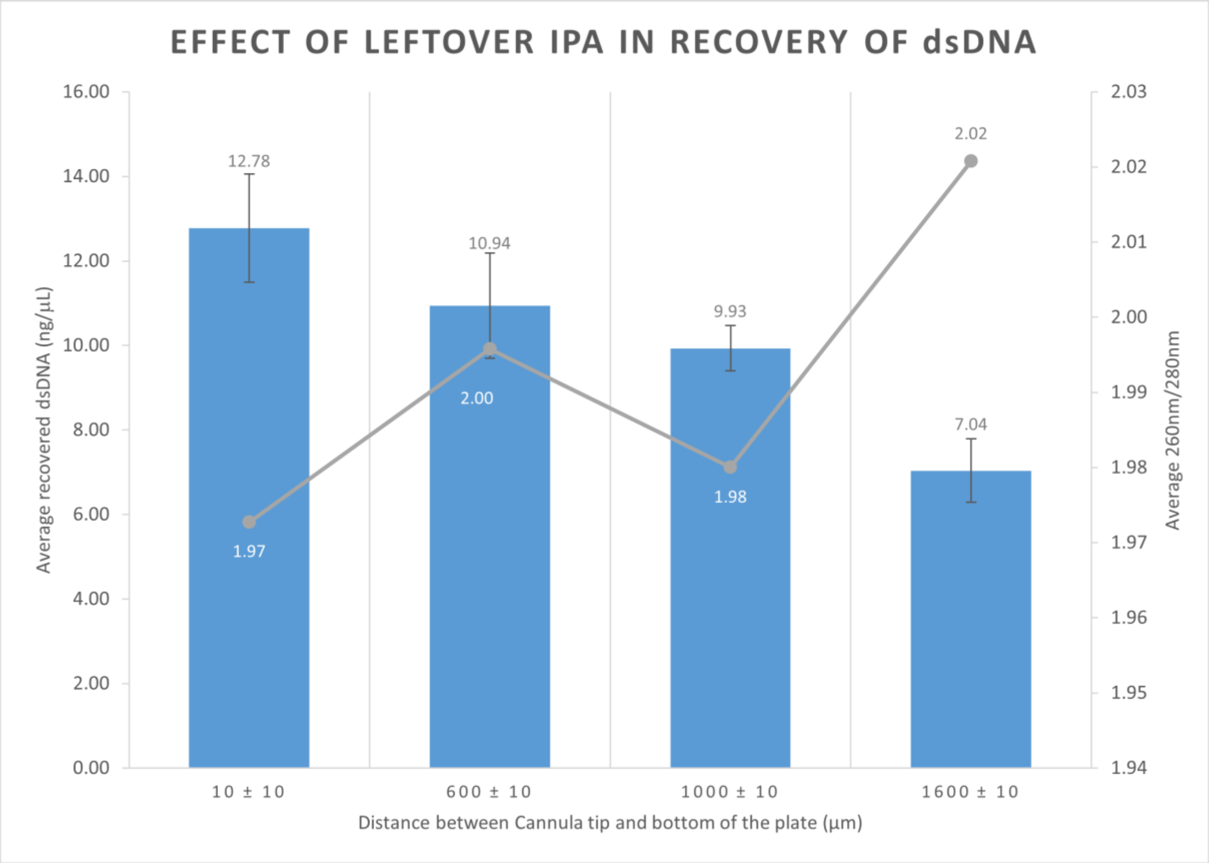

Figures S15. Effect of leftover isopropyl alcohol (IPA) during bead washing step on recovery of dsDNA. The leftover volume directly correlates with the distance between the cannula tip and the 384-well plate bottom. Additional IPA lowers dsDNA yield, but the average 260nm/280nm absorbance stays relatively consistent.

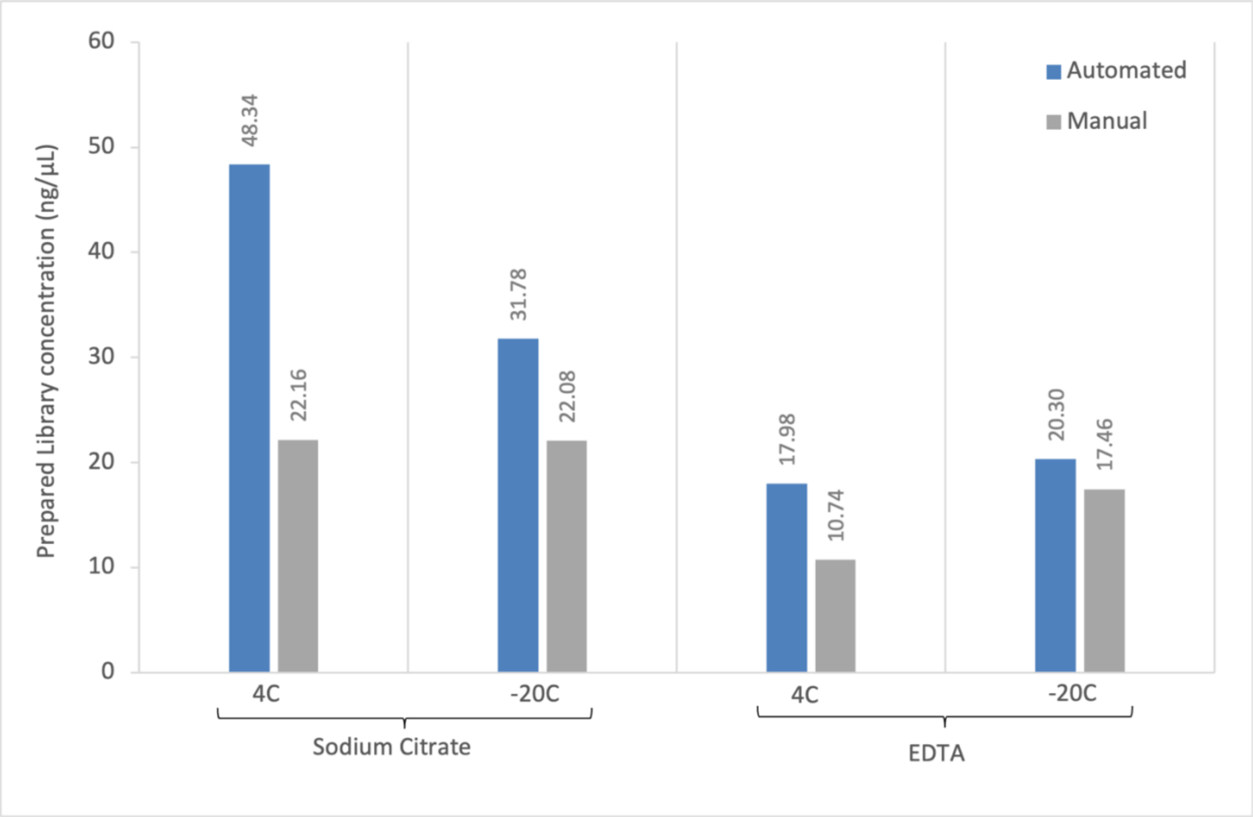

Figure S16. The measured concentration of the prepared libraries using automated and manual workflows.
